# From Germ Cell Histogenesis to Precision Risk Stratification: Prognostic and Predictive Molecular Markers in Testicular Germ Cell Tumors—A Systematic Review

**DOI:** 10.3390/medsci14060633

**Published:** 2026-10-02

**Authors:** Andrei Bogdan Cutitaru, Irina Draga Caruntu, Raluca Anca Balan, Carmen Solcan, Cornelia Amalinei

**Affiliations:** 1 Department of Morphofunctional Sciences I, Grigore T. Popa University of Medicine and Pharmacy Iasi, 700115 Iasi, Romania; irina.caruntu@umfiasi.ro (I.D.C.); raluca.balan@umfiasi.ro (R.A.B.); cornelia.amalinei@umfiasi.ro (C.A.); 2 Faculty of Veterinary Medicine, Ion Ionescu de la Brad University of Life Sciences, Aleea Mihail Sadoveanu No. 3, 700490 Iasi, Romania; carmen.solcan@iuls.ro

**Keywords:** germ cell neoplasia in situ, microRNA, liquid biopsy, pluripotency transcription factors, risk stratification

## Abstract

**Background/Objectives:** Testicular germ cell tumors (TGCTs) represent the most common solid malignancies in young adult men, with cure rates exceeding 95% under modern cisplatin-based therapy, yet a clinically relevant subset of patients still experiences treatment failure or long-term toxicity. This systematic review synthesizes peer-reviewed evidence, from database inception through 6 July 2026, on prognostic and predictive molecular markers in developmental and endocrine-sensitive testicular carcinogenesis. **Methods:** A PRISMA 2020-aligned search of MEDLINE/PubMed, Embase, Web of Science Core Collection, and the Cochrane Library was supplemented by registry-based evidence, reference-list screening, and contemporary guidelines to capture diagnostic, prognostic, and predictive biomarkers in TGCTs. The strategy encompassed classical serum markers, tissue-based molecular and immunohistochemical markers, miRNA/cfDNA liquid-biopsy assays, immune and resistance-related features, and validated clinicopathological risk models. **Results:** The review integrates classical serum biomarkers, pluripotency transcription factors, surface and oncofetal antigens, yolk-sac and trophoblastic markers, circulating microRNAs, methylated cell-free DNA, immune features, and updated clinicopathological risk models. Markers are graded as established, validated research, or emerging candidates. miR-371a-3p shows a high accuracy for active non-teratomatous TGCT, with pooled sensitivity of about 0.90 and specificity of approximately 0.86, but is negative in pure teratoma. AFP, β-hCG, and LDH are still required for staging and IGCCCG risk assignment. **Conclusions:** Despite the growing promise of biomarker-driven approaches in TGCT, no single serum, tissue, liquid-biopsy, immune, or resistance-associated marker has yet demonstrated sufficient clinical utility to replace comprehensive evaluation based on morphology, staging, histology, imaging, standard serum markers, and established clinicopathological risk assessment.

## 1. Introduction

In young adult men, TGCTs represent a distinct clinicopathological entity, combining the highest incidence among solid malignancies in this age group with cure and survival rates exceeding 95% after contemporary cisplatin-based treatment [1,2,3]. Nevertheless, a minority of patients relapse or endure significant long-term treatment-related morbidity, motivating interest in prognostic and predictive molecular markers that refine risk stratification beyond conventional clinicopathological factors [1,2,3,4,5].

Developmentally linked and endocrine-disruption-associated testicular carcinogenesis is firmly linked to disturbances in fetal and perinatal gonadal development, with germ cell neoplasia in situ (GCNIS) considered the precursor of nearly all post-pubertal seminomas and non-seminomatous germ cell tumors (NSGCTs) [6,7,8]. The 5th edition of the WHO Classification of Tumours of the Urinary and Male Genital System (2022) confirms GCNIS as the standard term for the intratubular precursor, places seminoma within the broader “germinoma” family alongside its extragonadal counterparts, and explicitly distinguishes GCNIS-derived TGCTs from the prepubertal-type teratoma and the spermatocytic tumor, which arises from post-pubertal spermatogonia and is biologically and prognostically distinct [9,10,11]. The term “spermatocytic seminoma” is therefore obsolete and has been replaced by “spermatocytic tumor”.

This review synthesizes peer-reviewed evidence on prognostic and predictive molecular markers in developmental and endocrine-sensitive testicular carcinogenesis, integrating classical serum markers, pluripotency-related transcription factors, surface receptors, oncofetal antigens, epigenetic and microRNA signatures, immune and microenvironmental markers, and validated risk models and nomograms. Particular emphasis is placed on markers expressed in both normal testicular histogenesis and malignant germ cell clones; on the explicit grading of clinical evidence; and on updated guideline frameworks, including IGCCCG Update 2021, WHO 2022, EAU 2023, and SEOM-GG 2023 [9,12,13,14,15,16].

The primary objective of our systematic review was to critically evaluate the prognostic and predictive molecular markers in developmental and endocrine-sensitive testicular carcinogenesis, and to classify TGCTs according to their current level of clinical applicability. Secondary objectives were to relate candidate biomarkers to TGCT histogenesis and the WHO 2022 classification; to compare emerging markers with established serum and clinicopathological risk factors; and to identify evidence gaps that currently prevent their routine implementation in risk stratification, treatment selection, and surveillance.

## 2. Materials and Methods

This systematic review was reported in accordance with the Preferred Reporting Items for Systematic Reviews and Meta-Analyses (PRISMA) 2020 statement (Supplementary Materials). The review was not registered, and no separate review protocol was prepared.

### 2.1. PICO Framework

In the context of the present systematic review, the PICO framework was used to translate the manuscript objective into an explicit evidence question: which molecular, immunohistochemical, serum, and liquid-biopsy markers provide prognostic or predictive information in TGCTs beyond conventional clinicopathological assessment? The population (P) comprised patients with TGCTs (seminomas and NSGCTs) or GCNIS, together with experimental models reflecting developmental and endocrine-sensitive testicular carcinogenesis [1,8]. The intervention or exposure (I) was the assessment of molecular, immunohistochemical (IHC), serum, or liquid-biopsy biomarkers, including transcription factors, surface markers, genomic, epigenetic, and microRNA signatures, with presumed prognostic or predictive value [4,15,17]. The comparator (C) was the conventional clinicopathological factors, the standard serum markers (AFP, β-hCG, and LDH), or the absence/low expression of the candidate biomarker, with healthy or benign testicular tissue serving as the reference in translational studies [18,19]. Outcomes (O) included diagnostic classification, stage at presentation, progression-free survival (PFS), risk of relapse, overall survival (OS), therapy response (particularly to cisplatin-based chemotherapy and salvage regimens), and risk-group assignment using the International Germ Cell Cancer Collaborative Group (IGCCCG) and related models [12,13,14].

### 2.2. Search Strategy and Study Selection

A systematic search was conducted in MEDLINE/PubMed, Embase, and Web of Science Core Collection from database inception through 6 July 2026, with the Cochrane Library used as an additional source for systematic-review cross-checking. The search combined controlled vocabulary and free-text terms for “testicular germ cell tumor”, “seminoma”, “non-seminomatous germ cell tumor”, “germ cell neoplasia in situ”, “biomarker”, “prognostic”, “predictive”, “serum marker”, “microRNA”, “miR-371a-3p”, “M371”, “OCT4”, “NANOG”, “SALL4”, “SOX17”, “KIT”, “RASSF1A”, “risk model”, “nomogram”, “liquid biopsy”, “cfDNA”, “methylation”, “immune microenvironment”, and “cisplatin resistance”; topic-specific sub-searches covered miR-371a-3p/miRNA liquid biopsy, cfDNA/RASSF1A methylation, IHC pluripotency markers, immune microenvironment, genomics/transcriptomics, cisplatin resistance, residual disease/RPLND, and stage I surveillance. Reference lists of eligible articles, key reviews, guidelines, and registry-based studies were also screened, including the IGCCCG Update Consortium, EAU 2026 testicular cancer guidance, SEOM-GG 2023, and SEER [12,13,16,20,21].

Eligibility was a priori defined by using the PICO framework. Eligible population comprised patients with TGCTs (seminomas or NSGCTs) or GCNIS and directly relevant translational or experimental models of testicular germ cell carcinogenesis. Reports had to evaluate at least a molecular, immunohistochemical, serum, genomic or epigenetic, microRNA, liquid-biopsy, immune, resistance-related, or clinicopathological marker and to provide an extractable diagnostic, prognostic, predictive, monitoring, treatment-response, relapse or survival, staging, or risk-classification outcome. Eligible evidence included primary clinical or translational studies; diagnostic-accuracy studies; randomized or observational cohorts; registry or modeling analyses; systematic reviews and meta-analyses; and authoritative guidelines, consensus, or classification sources. Narrative reviews were only used for biological context or for the reference-list identification. The main exclusions were the following: non-TGCT cases without a relevant comparator, no marker–outcome relationship or extractable data, therapy-only reports without a marker endpoint, insufficient publication data or bibliographic information, duplicate or overlapping reports (retaining the most informative synthesis where appropriate), and non-human or laboratory-only studies without a direct TGCT translational link. In addition, full text had to be accessible and readable in the original language or translation. Reports identified through database screening and supplementary or other methods were assessed against the same eligibility framework.

**Figure 1:**
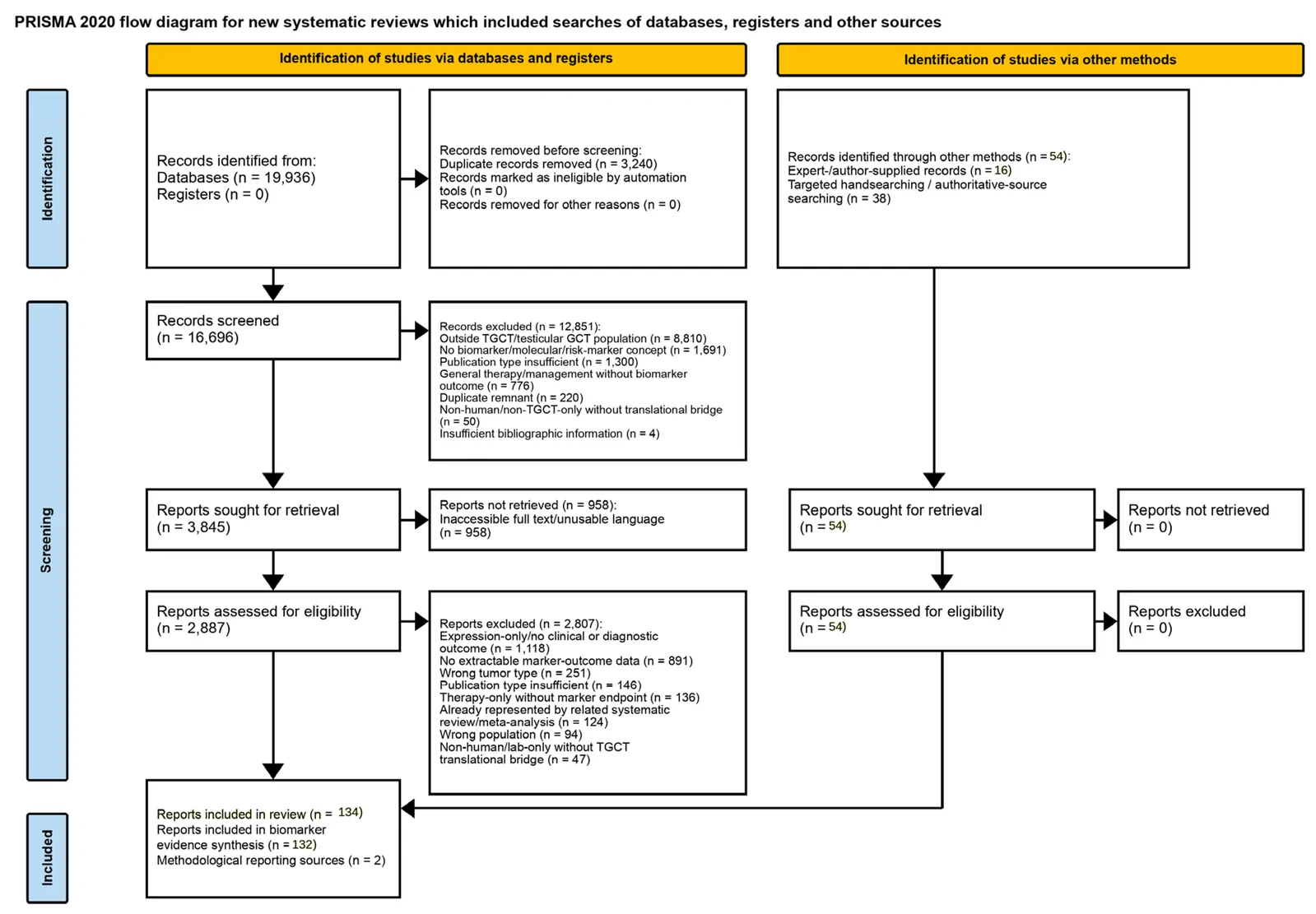
PRISMA 2020 flow diagram of the study selection process. Diagram structure adapted from the PRISMA 2020 flow diagram template (https://www.prisma-statement.org/prisma-2020-flow-diagram (accessed on 18 July 2026)) and Page et al. [22], licensed under the Creative Commons Attribution 4.0 International License (CC BY 4.0; https://creativecommons.org/licenses/by/4.0/ (accessed on 18 July 2026)). Abbreviations: PRISMA—Preferred Reporting Items for Systematic Reviews and Meta-Analyses; TGCT—testicular germ cell tumor; GCT—germ cell tumor.

The electronic database search identified 19,936 source-level records before cross-database de-duplication, comprising 4921 MEDLINE/PubMed records, 12,739 Embase records, and 2276 Web of Science Core Collection records. De-duplication removed 3240 duplicate records across 2656 duplicate groups, leaving 16,696 unique records for title/abstract screening; 12,851 records were excluded and 3845 reports were sought for retrieval. Of these, 958 reports were not retrieved, 2887 reports were assessed for eligibility, and 2807 full-text reports were excluded with primary reasons. The database-screening route yielded 80 included reports. Additional reports identified through other methods (*n* = 54) comprised expert-/author-supplied reports (*n* = 16) and targeted handsearching/authoritative-source searching (*n* = 38). In total, 134 final reports were included in the review, comprising 132 biomarker evidence-synthesis reports and 2 methodological reporting sources. Reporting was aligned with PRISMA 2020 [22] (Supplementary Materials) and is summarized in the study-selection workflow diagram (Figure 1). The term “included reports” refers to publications that met the predefined PICO-based eligibility criteria after systematic title/abstract and full-text screening; given the heterogeneity of the reports, a narrative synthesis was performed instead of a de novo quantitative meta-analysis.

The Cochrane Library cross-check identified 0 Cochrane Reviews, 0 Cochrane Protocols, and 36 CENTRAL/Trials records; the CENTRAL/Trials set was retained as a count-only cross-check and was not imported into the de-duplicated bibliographic pool because record-level export was not available.

### 2.3. Data Extraction and Evidence Grading

For each eligible study, data were extracted on design, sample size, TGCT subtype and stage, biomarker type and assay, cut-offs, reference comparators, and prognostic or predictive endpoints. Given the high heterogeneity in assay platforms and endpoints, a narrative synthesis was favored over a new pooled meta-analysis, but results were integrated with recent meta-analyses and large registry studies whenever available—particularly for microRNAs, RPLND/residual-disease pathology, and clinical risk factors [23,24,25,26,27]. Pooled sensitivity, specificity, and diagnostic odds ratio values were extracted from previously published systematic reviews and meta-analyses and descriptively used to complement the narrative synthesis. The only marker/outcome domains with sufficient extractable diagnostic-test data, particularly miR-371a-3p for active non-teratomatous TGCT and for residual/RPLND viable disease, are already covered by recent systematic reviews and meta-analyses, whereas ctDNA, methylation, immune/IHC, risk-model, and cisplatin-resistance studies use heterogeneous assays, thresholds, endpoints, and study designs. The synthesis therefore incorporates available pooled estimates from recent meta-analyses and adds a formal feasibility assessment, treating non-teratomatous miR-371a-3p applications as potentially meta-analyzable in a dedicated review and teratoma, exploratory ctDNA/methylation, IHC, and resistance mechanisms as unsuitable for new aggregate pooling at this stage.

**Figure 2:**
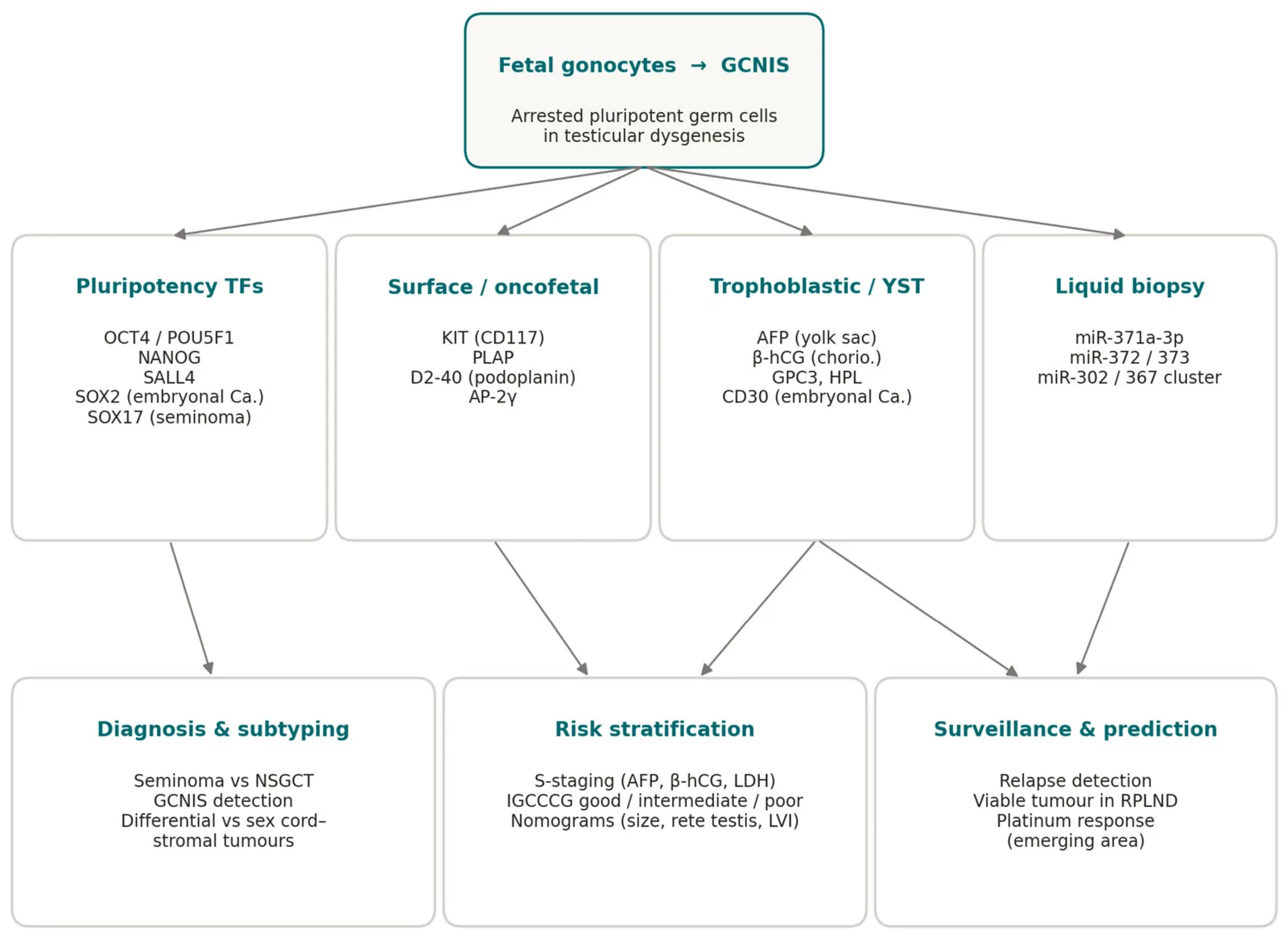
Conceptual map of the major classes of molecular markers in developmental and endocrine-sensitive testicular carcinogenesis. The diagram links the developmental precursor (germ cell neoplasia in situ, GCNIS) to four marker classes—pluripotency transcription factors (OCT4/POU5F1, NANOG, SALL4, and SOX2 in embryonal carcinoma and SOX17 in seminoma), surface and oncofetal antigens (KIT/CD117, PLAP, D2-40, and AP-2γ), trophoblastic and yolk-sac markers (AFP, β-hCG, GPC3, and HPL), embryonal-carcinoma marker CD30, and liquid-biopsy microRNAs (the miR-302/367 cluster and miR-371a-3p)—and to their main clinical applications in diagnosis and subtyping, IGCCCG-based risk stratification, and surveillance/response prediction. Original figure created by the authors; no copyrighted third-party material was reproduced.

**Figure 3:**
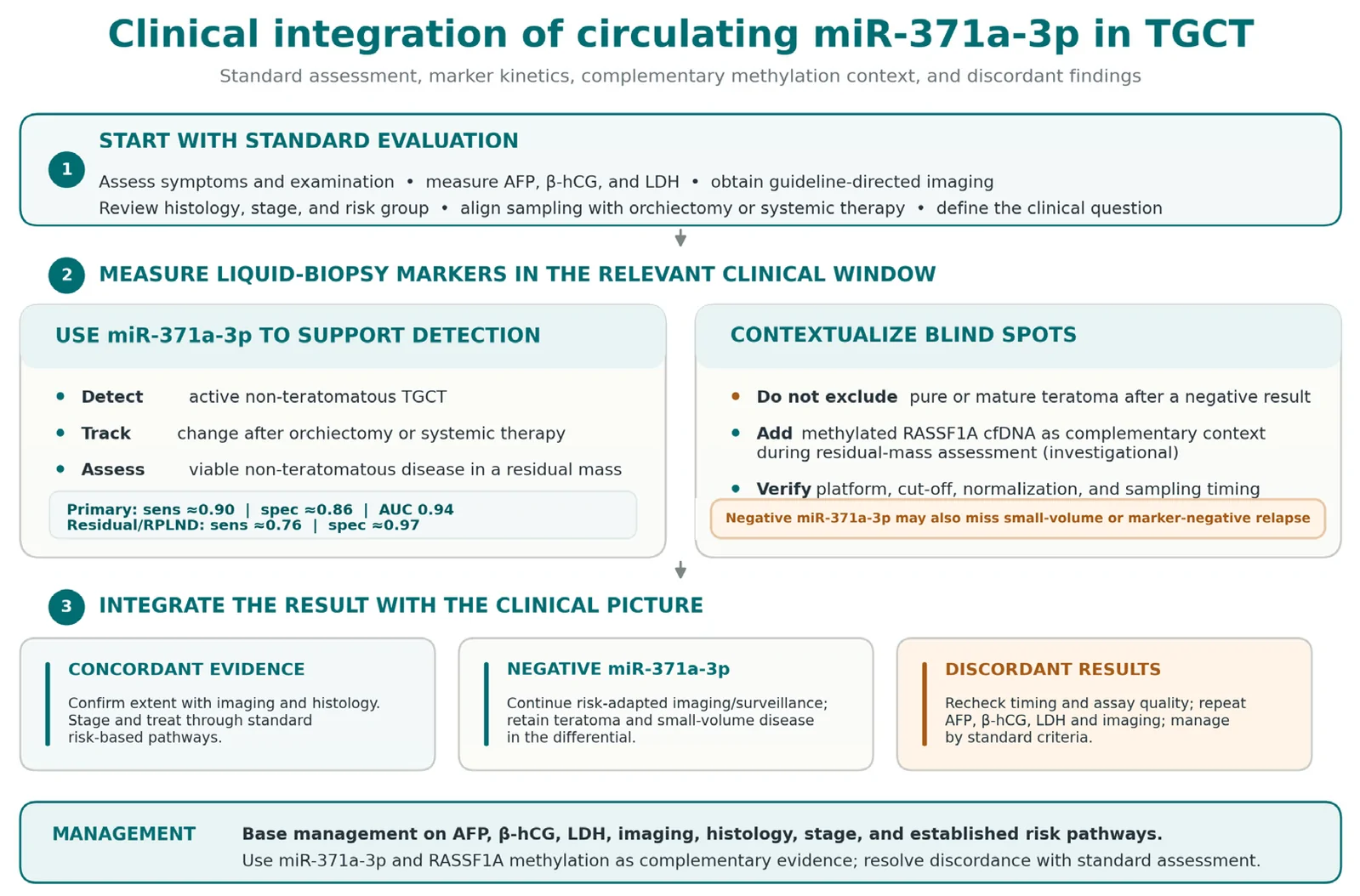
Clinical utility and limitations of circulating miR-371a-3p in TGCT. The figure summarizes diagnostic and residual-disease performance, potential applications in diagnosis and monitoring of active non-teratomatous disease; the teratoma blind spot and assay-standardization needs; and the complementary roles of methylated RASSF1A cfDNA, classical serum markers, histology, and imaging. Original figure created by the authors; no copyrighted third-party material was reproduced.

**Figure 4:**
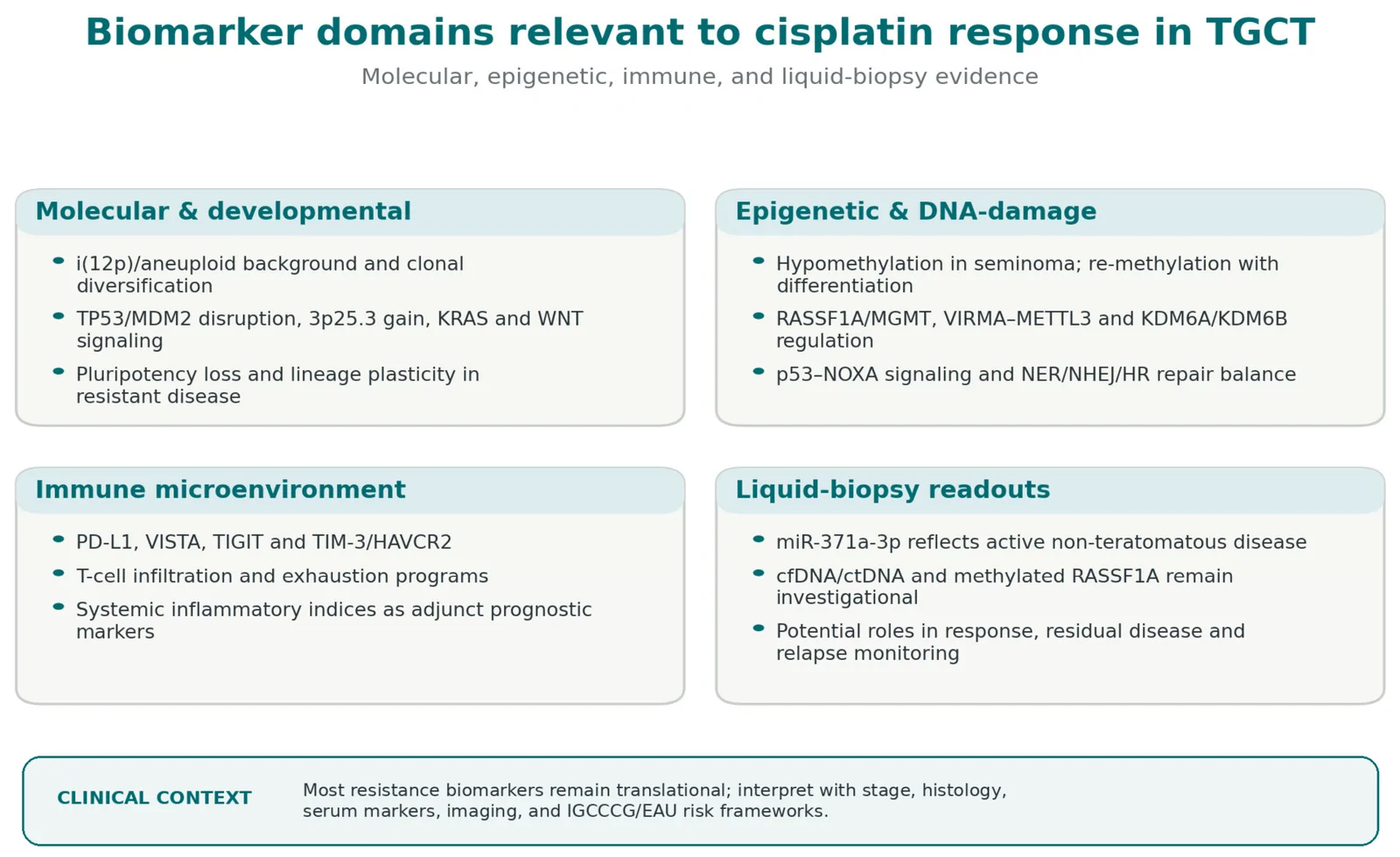
Biomarker domains relevant to cisplatin sensitivity and resistance in TGCT. Molecular and developmental, epigenetic and DNA-damage, immune, and liquid-biopsy findings are summarized together with their principal clinical relevance, including the current translational candidates and the continued importance of clinicopathological assessment. Original figure created by the authors; no copyrighted third-party material was reproduced.

Markers were explicitly graded into three tiers: (i) established clinical markers, in routine use and incorporated into guidelines; (ii) validated research markers, with multicenter evidence approaching clinical implementation; and (iii) emerging or unvalidated markers, supported mainly by single-center or translational data (Table 1). The major marker classes considered are summarized conceptually in Figure 2, the evidence synthesis is summarized in Table 2, the clinical implementation pathway and limitations of miR-371a-3p are illustrated in Figure 3, and mechanism classes relevant to cisplatin sensitivity and resistance are integrated in Figure 4.

## 3. Results

The results of our search regarding current TGCTs data have been organized into several evidence-based subsections for all the findings, according to the current knowledge and concepts, limitations, and future perspectives.

### 3.1. Biological Background: Developmental and Endocrine-Sensitive Carcinogenesis

Testicular carcinogenesis is anchored in the biology of primordial germ cells (PGCs) and gonocytes, where physiological expression of pluripotency factors (OCT3/4/POU5F1, NANOG, and SALL4) is required for early development but they must be downregulated as maturation progresses. The failure of this developmental switch in the dysgenetic testis determines GCNIS and subsequent invasive TGCTs [6,7]. Histopathological and experimental studies show that OCT4-positive/MAGEA4-negative gonocytes persisting beyond normal developmental windows are the most proliferative and invasion-prone population within intratubular neoplasia, supporting their role as precursors of seminoma and embryonal carcinoma [6,7,8,63].

Considering that the testis is both an endocrine target and a hormone-producing organ, hypothalamic–pituitary–gonadal axis perturbations, testicular dysgenesis syndrome (TDS), cryptorchidism, and prenatal exposure to endocrine-disrupting chemicals (phthalates and bisphenols) are established risk factors for TGCTs, probably acting through epigenetic disruption of fetal gonocyte maturation [64,65].

A notable feature is the existence of shared regulatory pathways between histogenesis and carcinogenesis, which include pluripotency networks (OCT3/4–SOX2/SOX17–NANOG–SALL4), growth-factor signaling (KIT/KITLG, RAS/ERK, PI3K/AKT, and WNT), and microenvironmental cues (Sertoli, Leydig, peritubular cells, and immune infiltrates) [8,66,67]. The involvement of these networks is reflected in the expression profiles of many clinically relevant biomarkers, defining a morphology-led carcinogenesis model supported by molecular evidence [1,8,56,67].

Although many data are currently available, the precise timing and reversibility of the developmental switch are incompletely defined in humans, and prospective data linking specific endocrine-disruptor exposures to individual biomarker expression are still lacking.

### 3.2. International Registries and Risk-Classification Systems

Risk classification in TGCTs relies on population-level data and validated prognostic models that stratify patients for treatment and surveillance.

Large cancer registries, such as SEER and European national datasets, provide reliable estimates of TGCTs incidence, stage distribution, and survival, serving as platforms for the validation of prognostic factors. SEER data confirm that testicular cancer accounts for about 1% of male cancers but represents the most common malignancy in men aged 15–40 years, with an age-adjusted incidence of about 6 per 100,000 and mortality of around 0.3 per 100,000 in recent years [20]. European registries similarly document high cure rates but rising incidence, particularly of seminoma, in Northern Europe, with a 1–2% annual increase in Western countries, underlining the need for refined risk stratification to avoid over- or undertreatment [3,68,69].

The IGCCCG classification, based on primary tumor site, the detection of non-pulmonary visceral metastases, and pre-chemotherapy levels of AFP, β-hCG, and LDH, is still the cornerstone for assigning patients with metastatic TGCTs to good-, intermediate-, and poor-prognosis groups [12,13]. The IGCCCG Update Consortium, drawing on 9728 patients with metastatic NSGCTs, along with 2451 patients with metastatic seminoma diagnosed and treated between 1990 and 2013, confirmed the robustness of this model but documented substantially improved survival relative to the original 1997 cohort and identified additional adverse factors, most notably LDH ≥ 2.5 × upper limit of normal (ULN) in the good-prognosis group, together with increasing age and lung metastases in NSGCTs [12,13]. An online calculator (eortc.org/IGCCCG-Update) implements the updated model.

These registry-derived systems constitute the clinical baseline used to benchmark every candidate molecular biomarker. Updated EAU and SEOM-GG guidelines incorporate these data and acknowledge that prognostic-factor performance may differ between high-volume expert centers and lower-volume settings [16,70,71].

Misclassification may occur if pre-orchidectomy rather than pre-chemotherapy marker levels are used, leading to clinically consequential up- or downstaging in 8–22% of metastatic patients and inappropriate escalation or de-escalation of therapy [33,72]; integrating molecular markers into these registry frameworks is still an open task (Table 3).

### 3.3. Classical Serum Markers

Among the classical serum markers, AFP, β-hCG, and LDH are the established serum biomarkers integrated into TNM “S” staging and IGCCCG risk classification (Table 4).

A significant notion is that of the S category that is used to group the patients according to specific post-orchidectomy tumor markers’ levels, with S0 indicating normal values and S1–S3 defining increasing degrees of elevation that correlate with tumor burden and prognosis. NSGCTs—particularly those with embryonal carcinoma, yolk-sac, or choriocarcinoma components—secrete AFP and/or β-hCG in most cases, whereas pure seminoma, by definition, does not produce AFP. Therefore, persistent post-orchidectomy AFP elevation rules out a pure-seminoma diagnosis. LDH is a less specific but burden-reflective marker in both histologies [18,19].

It is important to acknowledge that pre-orchidectomy and pre-chemotherapy marker kinetics and half-lives (approximately 5–7 days for AFP, 1–3 days for β-hCG, and ~24 h for LDH) are critical for accurate staging, early detection of residual disease, and appropriate IGCCCG assignment [72,73]. Another mention is mandatory, regarding the use of pre-chemotherapy values, not of pre-orchidectomy values, for IGCCCG classification [33,72].

Although traditional markers are essential, they have limited sensitivity and specificity for relapse detection in clinical stage I (CS1) NSGCTs under surveillance [35]. A PRISMA-based systematic review of 37 studies in CS1 NSGCTs reported that only 28–75% of relapses presented with elevated markers, while serum markers alone, without imaging evidence, detected relapse in only 4–39% of cases [35]. Combined AFP, β-hCG, and LDH sensitivity for TGCT diagnosis is therefore approximately 50–65%, and up to 70% of CS1 seminoma patients are marker-negative at orchidectomy [26,74].

These limitations in biomarker utility motivate the development of complementary microRNA- and ctDNA-based tools able to detect disease when classical markers are negative.

### 3.4. Pluripotency-Related Transcription Factors

Pluripotency transcription factors reprise their developmental roles in GCNIS-derived tumors and form the core of the diagnostic IHC panel (Table 5).

An important finding regarding OCT3/4/POU5F1, a central regulator of pluripotency, is that it exhibits strong nuclear expression in GCNIS, classical seminoma, and embryonal carcinoma but its expression is absent in normal postnatal spermatogonia and most somatically differentiated teratomatous elements [19,75]. It has been demonstrated that prolonged OCT4 expression in germ cells is closely associated with malignant transformation. As noted in Section 3.1, OCT4+/MAGEA4- GCNIS cells are the most proliferative and invasion-prone, supporting a biological gradient transition from intratubular neoplasia to invasive seminoma and NSGCTs [6,7,63]. Additionally, large IHC series confirm that OCT3/4 is an exceptionally sensitive and specific marker for seminoma and embryonal carcinoma [1,15,75].

Another specific biomarker is SALL4, a broad marker of GCNIS-derived GCTs (seminoma, embryonal carcinoma, and yolk-sac tumor), exhibiting a characteristic loss of expression in many differentiated teratoma components [15,19,30]. Conversely, NANOG shows intense nuclear positivity in GCNIS, seminoma, and embryonal carcinoma but is largely negative in differentiated teratoma tissues [1,15].

Other specific markers, such as SOX2 and SOX17, two members of the SOX transcription-factor family, show a diagnostically useful complementary distribution. SOX2 is characteristically expressed in embryonal carcinoma but negative/low in seminoma [19,76]. Conversely, SOX17, an endodermal lineage factor, functionally replaces SOX2 in seminoma and GCNIS, binding canonical OCT4/SOX2 motifs and driving TFAP2C, PRDM1, and PRDM14 [31]. Moreover, a recent study showed that SOX17 induction, together with WNT, TGF-β, and FGF signaling, can divert embryonal carcinoma cells toward a yolk-sac-tumor phenotype with upregulation of FOXA2, GATA6, and GPC3 and acquisition of cisplatin resistance [77].

Recently, PRAME has emerged as an additional seminoma marker, with reported sensitivity of approximately 92% and specificity of around 92%, potentially superior to SOX17 alone in distinguishing seminoma from non-germ cell mimics [78].

These marker characteristics primarily support histological classification and subtyping rather than independent prognostication, reinforcing the morphological categories defined in the WHO 2022 classification. However, assay standardization, antibody clones, and interpretation of variable or component-dependent staining still limit their reproducibility, and none of these markers has independent prognostic value beyond that of recognized clinicopathological factors.

### 3.5. Surface Receptors and Oncofetal Antigens

Surface receptors and oncofetal antigens are added to the pluripotency markers and may clarify different mechanistic questions, such as KIT marker role in testicular carcinogenesis.

KIT (CD117), a type III receptor tyrosine kinase, is strongly expressed on fetal gonocytes and GCNIS cells, showing diffuse membranous positivity in most seminomas (approximately 90–97% membrane positivity), with markedly lower rates in non-seminomas [1,4,15].

Additionally, podoplanin (D2-40), AP-2γ/TFAP2C, and placental alkaline phosphatase (PLAP) are oncofetal antigens with high sensitivity for GCNIS and seminoma [19,75]. PLAP lacks TGCT specificity and has been largely supplanted by OCT3/4 in contemporary panels [75]. However, CD30 continues to serve as the key marker of embryonal carcinoma, with sensitivity in the order of 96% and high specificity [19].

It is important to distinguish two mechanistically different roles of the KIT/KITLG axis: (i) somatic KIT gain-of-function mutations, predominantly affecting codon 816 (D816H/V) in the activation loop, which are present in approximately 20–30% of seminomas and provide constitutive activation; (ii) common germline variants at KITLG and 5q31.3, which are the strongest known TGCT susceptibility loci on genome-wide association studies, with odds ratios of about 2.5 for variant rs995030 [67,79]. Furthermore, mutations of KRAS and NRAS, important downstream mediators of receptor tyrosine kinase and MAPK signaling, occur in 10–20% of seminomas [80].

KIT/CD117, PLAP, and D2-40/podoplanin are well-established diagnostic immunohistochemical markers for TGCT lineage assignment and subtype recognition, although their expression does not independently define prognosis [1,19]. In the context of somatic KIT gain-of-function mutations, it is important to note the association between molecular pathways and therapy resistance. A prime example is represented by the D816 mutation in the KIT tyrosine-kinase activation loop (encoded by exon 17), which determines primary resistance to imatinib. This resistance occurs because imatinib preferentially inhibits KIT receptors with exon 11 juxtamembrane-domain mutations, the canonical activating alterations seen in gastrointestinal stromal tumors, rather than exon 17 activation-loop mutants [67,81]. Because imatinib has shown no meaningful clinical activity in KIT-positive or refractory TGCTs, activating KIT mutations currently define a biologically distinct tumor subset rather than an actionable therapeutic target [67].

Regardless of their diagnostic value, component-dependent staining, particularly in mixed NSGCTs, and the absence of an effective KIT-directed therapy constitute the principal limitations for their practical utility.

### 3.6. Yolk-Sac, Trophoblastic, and Sex-Cord–Stromal Markers

Yolk-sac, trophoblastic, and sex-cord–stromal markers help identify divergent differentiation within testicular tumors, supporting the recognition of specific non-seminomatous germ cell components and the distinction between TGCTs and non-germ cell testicular neoplasms [9,19].

Their role is therefore primarily diagnostic and classification-oriented, especially when morphology alone does not clearly separate yolk-sac tumor, trophoblastic differentiation, embryonal carcinoma, seminoma, or sex-cord–stromal tumor.

Glypican-3 (GPC3) is a useful marker of yolk-sac tumor (YST) and is expressed in approximately 95% of YSTs, but its specificity is limited because staining may also occur in hepatoid teratoma components [19,82]. Because GPC3 is an oncofetal proteoglycan re-expressed during yolk-sac differentiation, it is most informative when interpreted together with morphology and additional lineage markers rather than in isolation.

Human placental lactogen (HPL) and β-hCG are expressed by syncytiotrophoblast cells and by intermediate trophoblast cells, with strong positivity in choriocarcinoma and in syncytiotrophoblastic cells within otherwise pure seminoma [19,83]. β-hCG is the more widely used trophoblastic marker and also functions as a serum biomarker, whereas HPL is a useful complementary marker for confirming trophoblastic differentiation when β-hCG staining is equivocal.

In addition, inhibin A is a sensitive marker for sex-cord–stromal tumors and is typically negative in seminoma and in most non-seminomatous germ cell tumor components, supporting its main use in separating sex-cord–stromal tumors from TGCTs [4,18]. Because inhibin A highlights Sertoli- and Leydig-cell derivatives, it is particularly valuable when a sex-cord–stromal tumor is considered in the differential diagnosis of a testicular mass.

Moreover, the panel of useful biomarkers has been completed by HNF1β, which has emerged as a diagnostically useful YST marker, with near-universal sensitivity reported in a tissue-microarray analysis of 601 TGCTs [19,29]. Compared with GPC3, HNF1β appears to provide superior specificity in distinguishing YST from embryonal carcinoma, particularly in mixed tumors, where overlapping morphology and immunophenotype may make interpretation more difficult [19,29].

Supplementing the panel of biomarkers, FOXA2 is an additional emerging YST-associated marker that may further refine the recognition of yolk-sac differentiation when used in combination with established markers [5,29]. As a forkhead box transcription factor active in endodermal differentiation, FOXA2 shows promise for identifying subtle or predominantly solid yolk-sac patterns that can be missed by GPC3 alone.

A practical diagnostic panel should include GPC3 and HNF1β for suspected YST differentiation, β-hCG and HPL for trophoblastic differentiation, and inhibin A when a sex-cord–stromal tumor is part of the differential diagnosis. HNF1β is particularly useful when GPC3 positivity is difficult to interpret because of its potential positive expression in hepatoid teratoma or other mixed tumor components [19,29]. However, trophoblastic-marker expression should be interpreted together with morphological features because syncytiotrophoblastic-cell positivity within seminoma is not sufficient to diagnose choriocarcinoma.

This panel of markers is valid as a set of diagnostic lineage tools rather than as independent prognostic or predictive biomarkers. Regarding their comparative diagnostic value, GPC3 is sensitive but not fully specific and inhibin A mainly addresses the differential diagnosis between TGCTs and sex-cord–stromal tumors. Nonetheless, trophoblastic markers require cautious interpretation in the context of IHC–morphological correlations. Future studies are required to clarify whether HNF1β, FOXA2, or combined yolk-sac/trophoblastic panels can improve reproducibility in mixed tumors and whether any of these markers have clinically meaningful associations with relapse risk, treatment response, or survival beyond their established diagnostic value.

### 3.7. MicroRNAs and Other Liquid-Biopsy Markers

Circulating embryonic microRNAs, and above all miR-371a-3p, are the most clinically advanced liquid-biopsy markers in TGCTs. Compared with tissue-based immunohistochemistry, which requires surgical or biopsy material and reflects a single spatial sample, liquid biopsy provides a minimally invasive, repeatable readout of active tumor burden that can be used for real-time monitoring across the disease course.

Among emerging biomarkers, microRNA-371a-3p (miR-371a-3p), a member of the embryonic miR-371–373 and miR-302/367 clusters, has demonstrated the most consistent diagnostic performance for detection of active germ cell malignancy [18,32]. Multiple retrospective and prospective cohorts have demonstrated that circulating miR-371a-3p is over-expressed in more than 90% of patients with seminoma or non-teratomatous NSGCTs, correlates with tumor volume, and normalizes rapidly (within 24–48 h) after orchidectomy or effective chemotherapy [25,26,84]. A large pooled analysis aggregating patient-level data across several institutions similarly demonstrated that miR-371a-3p alone may be sufficient for diagnosis, with an area under the curve (AUC) of 0.94, whereas other cluster members (e.g., miR-372/373/367) added no diagnostic value [32]. Pooled sensitivity and specificity across contemporary series are approximately 0.90 and 0.86, respectively [26].

The strongest historical validation is still provided by the prospective multicenter M371 study, which examined 616 patients with testicular GCTs compared with 258 controls and reported a sensitivity of 90.1%, a specificity of 94.0%, and an AUC of 0.966 for primary diagnosis, with rapid decline after treatment and absent expression in teratoma [18]. Recent small-mass and real-practice cohorts refine rather than overturn this conclusion. Specifically, miR-371a-3p had a sensitivity of 87% and a specificity of 91% in the best-performing plasma RT-qPCR workflow for small testicular masses, a sensitivity of 77.5% and a specificity of 80% in a CE-certified real-world cohort of small testicular masses, and substantially lower sensitivity in an unselected real-practice cfDNA and miRNA cohort, emphasizing workflow and population heterogeneity [46,47,48].

In the post-orchidectomy and post-chemotherapy setting, miR-371a-3p is promising for the detection of viable, non-teratomatous germ cell malignancy. In this regard, a systematic review and meta-analysis of the retroperitoneal lymph node dissection (RPLND) specimens, miR-371a-3p, showed pooled sensitivity and specificity of approximately 0.76 and 0.97 for identifying viable tumor, excluding pure teratoma, with a diagnostic odds ratio exceeding 30 [27]. Furthermore, prospective data from the CLIMATE (ANZUP 1906) trial in 200 CS1 patients showed that a detectable post-orchidectomy miR-371a-3p was associated with a hazard ratio of 10.3 for relapse, with 24-month recurrence-free survival of 32% versus 89% in those with negative results; the marker outperformed tumor size and lymphovascular invasion (LVI) for relapse prediction, with an AUC of 0.77–0.86, positive predictive value of 0.62, and negative predictive value of 0.91 [85].

For the assessment of residual disease and lymph-node dissection specimens, available evidence supports a similar conclusion. In this regard, a systematic review and meta-analysis restricted to RPLND pathology found high specificity for viable non-teratomatous tumor but substantially poorer performance when teratoma was counted as viable disease, and a chemotherapy-naïve lymph-node dissection series reported detectable M371 levels in 85% of seminoma metastases and in virtually all vital non-seminomatous tumors, whereas teratoma showed no comparable signal [27,49]. In addition, tumor-informed ctDNA and methylation-based cfDNA assays are promising complements, but current studies show heterogeneous sensitivity and limited teratoma detection, so they are still investigational rather than validated replacements for histology or imaging [36,50,86]. Early data also indicate that miR-371a-3p declines significantly during BEP chemotherapy and that persistently positive post-chemotherapy levels may predict early relapse [87]. Additional liquid-biopsy approaches under active investigation include circulating tumor DNA (ctDNA) and multi-analyte panels combining cfDNA with N-glycan analysis, which have shown AUC values around 0.96 for NSGCTs in early studies [17,37].

As a final remark, miR-371a-3p is best regarded as a validated research marker approaching clinical implementation for non-teratomatous active disease. The conceptual framework of miR-371a-3p applications and limitations is summarized in Figure 3; the diagnostic estimates shown in the schematic derive from pooled meta-analyses and the CLIMATE prospective cohort [26,32,85].

For clinical translation, miR-371a-3p and methylated RASSF1A can be operationalized as an adjunctive decision pathway, while physical assessment, AFP, β-hCG, LDH, histology, risk-group assignment, and cross-sectional imaging remain the primary determinants of management [16,21,73]. The following clinical algorithm summarizes the considerations discussed above:

- Clinical stage I active surveillance: after orchiectomy, establish a baseline with AFP; β-hCG; LDH; cross-sectional imaging; and, where locally validated, miR-371a-3p. Then, repeat conventional markers and imaging according to standard surveillance schedules. A rising or persistently detectable miR-371a-3p should prompt confirmatory repeat testing and reflex standard work-up, including cross-sectional imaging and multidisciplinary review, because it may indicate active non-teratomatous relapse; a negative miR-371a-3p lowers the probability of active non-teratomatous disease but does not exclude teratoma or marker-negative relapse. If imaging, pathology or primary-tumor composition raises concern for teratoma despite negative miR-371a-3p, methylated RASSF1A cfDNA testing may be considered as a complementary research assay rather than as a stand-alone trigger for treatment [26,32,34,85,88,89,90].
- Post-chemotherapy residual mass evaluation: if AFP, β-hCG, and LDH have normalized but cross-sectional imaging shows a residual mass, miR-371a-3p positivity supports suspicion of viable non-teratomatous GCT and should accelerate guideline-based review for salvage therapy, resection, or biopsy as clinically appropriate. Conversely, a negative miR-371a-3p does not rule out mature teratoma or necrosis/fibrosis; RASSF1A methylation may help interrogate the teratoma blind spot, but management remains determined by imaging characteristics, standard serum markers, surgical pathology and guideline-based indications for post-chemotherapy resection [16,21,27,49,50,88,89,90].

Thus, prospective validation, external quality control, and assay standardization are required before miR-371a-3p/RASSF1A algorithms can replace established surveillance or residual-mass pathways [34,51,90].

Critical limitations of serum miR-371a-3p must be acknowledged. To begin with, miR-371a-3p is not reliably detected in pure mature teratoma; therefore, post-chemotherapy residual teratoma may test miR-negative despite clinically meaningful disease [18,88,89]. Therefore, combining serum miR-371a-3p with droplet-digital-PCR detection of methylated RASSF1A cell-free DNA captured approximately 88% of teratoma-only cases missed by miR-371a-3p and reached near-complete combined sensitivity in a research cohort, although this approach still requires prospective clinical validation [35]. Another relevant limitation is that miR-371a-3p may be detectable in selected non-malignant settings, which limits its suitability for population screening. Finally, assay platforms, cut-offs, and normalization strategies are not yet fully standardized; a CE-certified raw-Cq cut-off, Cq < 28, has been validated as preferable to the older relative-quantification threshold, RQ > 15, for relapse surveillance, although confirmatory repeat testing and further prospective validation are still required [34].

The implementation barrier of serum miR-371a-3p testing is therefore increasingly pre-analytical rather than conceptual. In support of this assertion, an interim multicenter analysis of paired sample handling reported similar plasma and serum M371 performances, supporting simplified workflows but still requiring final validation of shipping, stability, normalization, and cut-off robustness before decentralized routine use [51].

### 3.8. Molecular Profiling, Epigenetics, and Cisplatin Resistance

Multi-omics profiling and epigenetic studies illuminate both TGCT biology and the mechanisms of cisplatin sensitivity and resistance, providing a molecular framework for the characteristic chemosensitivity of these tumors and its occasional loss.

Large-scale genomic and transcriptomic studies, including The Cancer Genome Atlas (TCGA) analysis of TGCTs and the 100,000 Genomes Project, have characterized recurrent chromosomal alterations (notably isochromosome 12p), somatic mutations, methylation patterns, and expression signatures across histological subtypes [8,38]. These integrative datasets confirm that pluripotency genes (POU5F1, NANOG, SOX2/SOX17, and SALL4), KIT/KITLG signaling, and specific epigenetic regulators are central to TGCT biology and that genomic-instability patterns are different in seminoma compared with NSGCTs [8,38,80]. However, germline whole-exome meta-analyses have not identified a single high-penetrance susceptibility gene, supporting a polygenic model [91].

Clinically integrated whole-exome and transcriptome profiling of precursor lesions, primary testicular and mediastinal GCTs, and serial chemoresistant metastases linked the canonical i(12p)/aneuploid background to recurrent chromosome-arm-level amplifications and reciprocal deletions, referred to as reciprocal loss of heterozygosity. Taylor-Weiner et al. [92] further described KRAS acquisition during progression from precursor to primary disease, uniform TP53 wild-type status in primary TGCTs, high mitochondrial priming on BH3 profiling in primary tumors, and additional reciprocal loss of heterozygosity with loss of NANOG and POU5F1 in chemoresistant teratomas or transformed carcinomas [92]. These data place platinum sensitivity in a developmental context: most primary TGCTs retain a pro-apoptotic, pluripotent state, whereas resistant progression is associated with clonal evolution, lineage maturation, and erosion of pluripotency.

Complementary sequencing of 180 cisplatin-treated advanced GCTs showed that TP53 alterations were confined to resistant tumors and were especially frequent among primary mediastinal NSGCTs, while TP53-pathway disruption, including MDM2 amplification, was associated with poor IGCCCG risk and retained adverse prognostic value beyond IGCCCG categories [93]. The same study identified potentially actionable alterations in 55% of cisplatin-resistant tumors, including RAC1 mutations, supporting broad sequencing in refractory disease as an exploratory route to target selection rather than as an established standard [93].

For male type II GCTs specifically, cisplatin-resistant cell-line models and clinical tumor series converged on chromosome 3p25.3 gain; this was detected in all resistant cell lines, its copy number being correlated with resistance, while it was enriched in relapsed or cisplatin-resistant tumors, and it predicted a shorter progression-free and overall survival independently of IGCCCG risk and TP53/MDM2 status, particularly in NSGCTs, excluding pure teratoma [94]. Age-spanning integrated analysis of extracranial GCTs from patients 0–24 years old connected type I and type II developmental categories with WNT-pathway activation through somatic mutation, copy-number alteration, and promoter methylation. WNT activation was associated with poor clinical outcomes, while the use of WNT inhibitors actively suppressed GCT cells in preclinical in vitro and in vivo models [95].

Microdissection-based genome and transcriptome profiling of 547 histological units further separated histological diversity from clonal architecture [96]. While genomic and histological heterogeneity were not correlated, the fetal developmental transcripts were retained across tissues, the chromosome 12p expression changes were shared, and a conserved sequence of whole-genome duplication followed by clonal diversification was observed across malignant GCTs, with developmental timing differing between prepubertal and post-pubertal disease [96]. Together, these findings support a model in which developmental state, copy-number evolution, p53-axis disruption, WNT signaling, and 3p25.3 gain refine the mechanistic risk stratification but remain predominantly investigational until prospectively validated alongside stage, histology, serum markers, and IGCCCG/EAU frameworks.

Seminoma exhibits genome-wide DNA hypomethylation that mirrors the primordial-germ-cell methylome and may underpin its characteristic cisplatin hypersensitivity [97]. In seminoma, such hypomethylation is also associated with HERV expression, increased type I interferon signaling, and CD8+ T-cell infiltrates, providing a plausible link between its epigenetic state and immune-rich microenvironment [98]. NSGCTs show progressive re-methylation with differentiation [99,100]. In this context, promoter hypermethylation of MGMT, RASSF1A, and other tumor-suppressor genes has been proposed as a prognostic and resistance biomarker, while methylated RASSF1A in cfDNA underpins one of the most promising liquid-biopsy combinations [35]. Additional resistance mechanisms include m^6^A RNA-methylation changes via the VIRMA/METTL3 axis, up-regulation of neddylation (a ubiquitin-like modification that regulates protein turnover and stress responses), PI3K/AKT/mTOR activation, and MDM2 stabilization with attenuation of p53 signaling [41,101,102,103].

Newer translational data support a model in which lineage plasticity, chromatin state, and altered DNA-damage response jointly shape the therapy resistance. In this context, SLUG/EMT signaling has been linked to cisplatin-resistant NTERA-2 models, with lower SLUG expression associated with longer median PFS in in silico survival analysis [52]. Preclinical TGCT models showed that inhibition of the KDM6A/KDM6B demethylase pathway, either pharmacologically with GSK-J4 or experimentally by gene knockdown, increased the response of both cisplatin-sensitive and cisplatin-resistant tumors to cisplatin [53]. In sequenced, genomically profiled germ cell tumors, platinum-resistance-associated alterations involving KRAS, TP53, KIT, and MDM2 were linked to lower predicted platinum-sensitivity scores in chemotherapy-naïve tumors; however, these molecular predictions could not be directly correlated with treatment response because clinical outcome data were not available [54].

TGCT-model data link the unusually strong cisplatin response to p53 hypersensitivity and high constitutive NOXA levels mediated by OCT4, with epigenetic state and comparatively reduced nucleotide-excision-repair capacity acting as broader susceptibility features [39,104,105]. By contrast, acquired resistance involves rebalancing of DNA-repair pathways, with suppression of the classical non-homologous end-joining pathway, which repairs DNA double-strand breaks by directly ligating the broken DNA ends, together with compensatory activation of homologous recombination, providing a rationale for PARP–inhibitor combinations [39,106]. Counter-intuitively, CRISPR studies suggest that POU5F1 and NANOG may actively promote cisplatin sensitivity in embryonal carcinoma cells; therefore, their loss during clonal evolution may itself contribute to chemoresistance [107]. This layer of evidence is biologically compelling but has not yet reached clinical maturity.

The accumulated data support that the therapy resistance biomarkers are still primarily translational candidates and require prospective validation before their clinical use. These interrelated mechanisms are summarized in Figure 4.

### 3.9. Immune Microenvironment and Systemic Inflammation

The tumor immune microenvironment and systemic inflammatory indices have been proposed as prognostic and predictive readouts in TGCTs. Compared with immunologically “hot” tumors, such as melanoma and non-small-cell lung cancer, TGCTs, and seminoma in particular, display a distinctive immune landscape that is rich in infiltrating lymphocytes yet only modestly responsive to checkpoint blockade, underscoring that immune-cell abundance alone does not predict immunotherapy benefit in this setting.

In this direction, single-cell multi-omics analyses have revealed at least 15 immune-cell subtypes in the seminoma tumor microenvironment, with TFAP2C-mediated invasion programs and spatial clustering of exhausted T cells adjacent to tumor cells [56]. PD-L1, a marker of adaptive immune resistance, is expressed on tumor and immune cells, with the highest levels in choriocarcinoma and decreasing intensity through embryonal carcinoma, yolk-sac tumor, and seminoma [42,108]. PD-L1 correlates with adverse features, although low PD-L1 is paradoxically associated with better PFS [42].

Moreover, systemic inflammatory indices, such as platelet-to-lymphocyte ratio (PLR), neutrophil-to-lymphocyte ratio (NLR), systemic immune–inflammation index (SII), and haemoglobin–albumin–lymphocyte–platelet (HALP) score, are associated with advanced stage and worse prognosis on univariate analysis [43,109]. Furthermore, the combined assessment of VISTA on tumor-associated immune cells and a high platelet-to-lymphocyte ratio identifies advanced GCTs with substantially worse outcome (hazard ratio 4.10 for event-free survival and 15.56 for OS in one cohort) [43].

In addition, the latest immune and transcriptomic studies extend this exploratory field without yet changing the clinical practice. HAVCR2/TIM-3 expression has been proposed as a favorable prognostic immune marker in seminoma in integrated transcriptomic and tissue-validation analyses [57]. In addition, single-cell and spatial transcriptomic work has identified a LINC00152-high TGCT cell population associated with lymph-node metastasis, advanced stage, poor prognosis, and T-cell exhaustion [58]. TNFSF10 has also been proposed as a prognostic and immunotherapy-relevant marker through combined single-cell and TCGA analyses [59]. Consistent with the limited clinical yield of these markers so far, a 2026 review of microRNAs and immunotherapy emphasizes that immune engagement in TGCTs has not yet translated into reliable responses to checkpoint inhibition in unselected patients [60].

Beyond immune-checkpoint blockade, chimeric antigen receptor (CAR) T-cell therapy represents a molecularly selected, antigen-directed approach in TGCTs. CAR-T cells recognize cell-surface antigens independently of major histocompatibility complex presentation [110]. In this respect, claudin-6 (CLDN6), an oncofetal tight-junction protein expressed in most GCTs but largely absent from healthy adult tissues, has emerged as the leading target [110,111]. In the phase I BNT211-01 program, CLDN6-directed CAR-T cells, administered alone or with a CLDN6-encoding RNA vaccine, produced early responses in heavily pre-treated CLDN6-positive solid tumors, including a GCT cohort, although the small, selected population and early-phase design preclude conclusions about durability or routine use [111]. Together with the emerging bispecific T-cell engagers and antibody–drug conjugates directed against tumor-associated antigens, these data place CAR-T therapy within biomarker-enriched immunotherapy rather than as an extension of unselected checkpoint blockade [110,111].

The discordance between immune infiltration and poor checkpoint-inhibitor efficacy is, therefore, likely multifactorial: spatially clustered exhausted T-cell states may limit cytotoxic function near tumor cells, the low somatic-mutation burden of TGCTs reduces neoantigen availability, and the testicular immune-privileged milieu may sustain tolerogenic signaling even after malignant transformation [8,56,58,60]. These observations support biomarker-enriched or combination strategies that pair checkpoint blockade with immune-priming, epigenetics, DNA-damage-response, or cellular-therapy approaches, rather than further unselected checkpoint-inhibitor monotherapy, but such approaches remain investigational in TGCTs [60,112].

This body of evidence is still largely hypothesis-generating because anti-PD-1 and anti-PD-L1 monotherapies have shown only modest activity in heavily pre-treated, refractory TGCTs [112], and inflammatory indices are not independent of IGCCCG classification in most multivariable analyses [113,114].

Moreover, post-orchidectomy SII has been correlated with LVI and pathological stage in CS1 GCT, and the HALP score has shown the highest discriminatory performance among inflammatory indices (AUC ~0.74), but heterogeneous scoring and the absence of external validation in large prospective cohorts are limiting their clinical usability [44,45].

### 3.10. Clinicopathological Risk Models and Nomograms

Integrated risk prediction combines histopathology features, serum markers, and a continuously increasing amount of molecular markers (Table 6 and Table 7).

An important signification for CS1 seminoma managed with surveillance or risk-adapted strategies is represented by the tumor size and the rete testis invasion, which have been the traditional key risk factors for occult metastases [116]. Moreover, LVI within the primary tumor is the most validated prognostic factor in CS1 NSGCT, with traditional relapse risks of approximately 50% in LVI-positive versus around 15% in LVI-negative patients on surveillance [117,118,119].

**Table 7:** Stage I NSGCT relapse-risk factors derived from current Danish national and EAU systematic evaluations, with the emerging role of post-orchidectomy circulating miR-371a-3p as a prognostic marker outperforming size and LVI in the CLIMATE prospective cohort.

Risk Factor	Effect on Relapse Risk	Evidence	Author/Years
lymphovascular invasion (LVI)	~50% relapse vs. ~15% if absent	established (multiple cohorts)	Wagner et al. 2024; European Association of Urology 2026 [118,119]
embryonal carcinoma predominance (≥50%)	independent predictor after adjustment for LVI	validated (Danish 2024 series)	Wagner et al. 2024 [118]
predominant yolk-sac tumor	trend toward higher risk	inconsistent	European Association of Urology 2026 [119]
rete testis invasion/ T-stage	modest increase in risk	inconsistent	European Association of Urology 2026 [119]
tumor size and testicular hilum soft-tissue invasion	increased risk; multivariable model components	validated	Wagner et al. 2024 [118]
post-orchidectomy miR-371a-3p positive	HR 10.3; 24-mo RFS 32% vs. 89%	prospective (CLIMATE 2026)	Tran et al. 2026 [85]

The EAU 2023 three-tier risk model, derived from 1016 CS1 seminoma patients’ evaluation, replaces the older binary tumor size/rete testis classification with a model incorporating tumor size strata (≤2 cm, 2–5 cm, and >5 cm), rete testis invasion, and LVI, defining very-low- to high-risk groups with 5-year relapse rates from approximately 8% to 44–45%; this model has been externally validated in the TRISST randomized trial cohort [14,115]. Additionally, the AJCC 8th-edition pT1a/pT1b subdivision at 3 cm is undergoing prospective validation. Furthermore, a Danish national cohort and a contemporary EAU guideline evaluation identified embryonal carcinoma as an independent relapse predictor after adjustment for LVI [118,119].

Considering the risk variability, independent current assessments support multivariable risk groups but also highlight the calibration uncertainty in small validation cohorts. In this regard, a 2026 exploratory cohort found that DaTeCa and EAU clinical stage I seminoma risk groups have broadly aligned with observed relapse probabilities, but only rete testis infiltration was independently significant, while the EAU high-risk subgroup was very small [61]. Moreover, a Danish population-based machine learning model for stage I TGCT achieved moderate discrimination and ranked LVI, embryonal carcinoma, necrosis, rete testis invasion, tumor size, and LDH among the top predictors, supporting future model-building rather than immediate replacement of EAU/DaTeCa/IGCCCG frameworks [62].

Considering the risk prediction, this is inherently multivariable rather than single-marker. In support of this hypothesis, the prospective data from CLIMATE suggest that post-orchidectomy miR-371a-3p may outperform LVI and tumor size as a relapse predictor in CS1 disease and could subsequently refine these histopathological criteria [85,120]. However, the IGCCCG Update model is still the primary risk classifier in metastatic TGCTs. A more refined stratification by AFP, β-hCG, and LDH within the intermediate-prognosis NSGCT group reveals continuous outcome gradients, supporting their prognostic relevance and potential future refinement of risk models [121]. Nevertheless, these serum-marker-based systems do not fully capture teratoma-containing disease because mature teratoma is typically marker-negative and may persist as clinically relevant residual disease despite normal AFP, β-hCG, LDH, or miR-371a-3p levels [13,88]. Moreover, high-volume institutional experience and biological features such as teratoma in the primary tumor also influence PFS and OS [2,12,13,122].

Last but not least, external validation and formal clinical-utility studies are required before any molecular marker can replace components of the IGCCCG, TNM “S” staging, or EAU/SEOM-GG risk-stratification models.

### 3.11. Diagnostic Entities and Mechanism-Related Prognosis

Entity-specific marker profiles link morphology to prognosis across the TGCT spectrum, providing a practical bridge between histological diagnosis and clinical risk assessment.

In support of this assertion, a relatively homogeneous expression of pluripotency markers (OCT3/4, NANOG, SALL4, KIT, PLAP, D2-40, AP-2γ/TFAP2C, and SOX17) are registered in seminoma and its precursor GCNIS, reflecting their origin from arrested gonocytes [6,7,19]. In recognition of this characteristic, the ISUP consensus recommends a core diagnostic IHC panel of OCT3/4, PLAP, D2-40, SALL4, CD117, and CD30, complemented by detection of isochromosome 12p where indicated [19].

Additionally, PRAME may be added for additional seminoma specificity, particularly in extragonadal or metastatic settings [78]. In contrast, non-seminomas encompass embryonal carcinoma, yolk-sac tumor, choriocarcinoma, and teratoma—alone or in combination—and show more heterogeneous marker profiles, as follows: embryonal carcinoma is strongly positive for OCT3/4, NANOG, SALL4, SOX2, CD30, and cytokeratins; yolk-sac tumor expresses AFP, GPC3, HNF1β, SALL4, and cytokeratins; choriocarcinoma shows β-hCG, HPL, and GPC3 positivity; and teratomas often lack classical embryonal markers and serum miR-371a-3p expression, particularly in mature components [4,5,15,29].

More recently, immunophenotypic data in pure embryonal carcinoma and pure postpubertal-type yolk-sac tumor support the concept that GCNIS retains an OCT4/SOX17/NANOG phenotype and that reprogramming to non-seminoma occurs outside the spermatogonial niche rather than within GCNIS itself [123]. Moreover, CLDN6 expression appears common in GCNIS and non-teratomatous primary GCT but its expression is limited in post-chemotherapy teratoma, suggesting therapeutic relevance for non-teratomatous components but not a solution to mature teratoma detection [124].

From a prognostic standpoint, pure seminomas generally have an excellent outcome, but larger primary tumors (>5 cm), rete testis or hilar invasion, LVI, and elevated pre-chemotherapy β-hCG and LDH define subgroups with higher relapse risk in CS1 disease and potentially worse PFS in the metastatic setting [14,116,119]. Supplementary intratubular neoplasia must be considered in any discussion of entity-related prognosis because virtually all classical seminomas and most NSGCTs arise from GCNIS and retain GCNIS-like markers in invasive lesions [6].

It is worth noting that the spermatocytic tumors and other rare non-GCNIS-derived germ cell tumors lack the typical GCNIS markers (OCT3/4, PLAP, D2-40, and KIT), arise from post-pubertal spermatogonia, show distinct molecular profiles, and are not central to developmental carcinogenesis models dominated by GCNIS-derived seminoma and non-seminomatous tumors [9,11].

## 4. Discussion

Developmentally linked and endocrine-disruption-associated testicular carcinogenesis is orchestrated by developmental pathways that are reiterated in GCNIS and invasive TGCTs, with pluripotency factors (OCT3/4, NANOG, SALL4, and SOX2/SOX17), KIT/KITLG signaling, and oncofetal antigens representing key molecular hallmarks shared between histogenesis and carcinogenesis [1,6,31,56]. Moreover, the WHO 5th Edition classification (2022) consolidates GCNIS terminology and clearly separates GCNIS-derived seminoma and NSGCTs from the post-pubertal spermatocytic tumor, with direct implications for biomarker interpretation, given that the most useful diagnostic IHC markers are specifically positive in GCNIS-derived disease [9,11].

### 4.1. Developmental Histogenesis and Sexual Dimorphism in Germ Cell Carcinogenesis

Both TGCTs and ovarian germ cell tumors (OGCTs) originate from PGCs, whose migration, epigenetic reprogramming, and pluripotency maintenance are firmly regulated during embryogenesis. Accordingly, seminoma and ovarian dysgerminoma demonstrate striking molecular similarities, including persistent expression of OCT3/4, NANOG, SALL4, LIN28, KIT, and PLAP, reflecting the conservation of an undifferentiated germ cell phenotype rather than independent oncogenic programs [125,126]. Comparable pluripotency-associated transcriptional signatures in testicular and ovarian embryonal carcinoma further support developmental arrest as a framework for interpreting TGCT biology and biomarkers [125,126].

Despite these shared developmental origins, the biological context in which malignant transformation occurs varies significantly between the two sexes. In males, disruption of the fetal testicular niche and impaired gonocytes differentiation can promote the persistence of GCNIS, the obligate precursor of virtually all post-pubertal seminomas and NSGCTs [126]. No equivalent pre-invasive lesion is established in ovarian GCTs; this contrast emphasizes that the GCNIS-anchored pathway is male-specific [127]. Developmental differences between the gonads therefore provide context for distinguishing shared biomarker signals from TGCT-specific biology [125,126].

Additional molecular variations further emphasize the sexually dimorphic nature of germ cell carcinogenesis [128]. Gain of chromosome 12p, particularly isochromosome i(12p), is considered the genomic hallmark of post-pubertal TGCTs and represents one of the earliest events associated with malignant progression [129]. Its less consistent occurrence in ovarian GCTs reinforces the relevance of 12p gain to post-pubertal TGCT biology [128]. Differences in germ cell maturation and epigenetic reprogramming remain important developmental coordinates for biomarker expression interpretation [126]. Thus, epigenetic dysregulation, rather than a high burden of somatic mutations, remains central to the developmental model of germ cell tumorigenesis [128,129].

The close developmental analogy between seminoma and dysgerminoma extends beyond histology to clinical biomarkers. Serum AFP, β-hCG, PLAP, LDH, and the recently validated miR-371a-3p family reflect preserved germ cell biology and have demonstrated diagnostic or monitoring value across gonadal germ cell tumors [26,125,130]. Considering that most biomarker discovery studies are TGCT-based, ovarian findings should serve as a cautious external comparison rather than a basis for direct extrapolation [125,131].

Altogether, male and female GCTs share a primordial germ cell origin but diverge with gonadal differentiation [125,126,128]. For TGCT biomarkers, this comparison is most informative in the evaluation of conserved developmental markers, male-specific GCNIS pathways, and the significance of chromosome 12p [125,126,132].

### 4.2. Current Evidence Supporting Prognostic and Predictive Biomarkers in TGCTs

Compared with prior TGCT biomarker reviews that focus mainly on serum miRNAs, surgical staging, conventional serum markers, or individual molecular pathways, the present review demonstrates that prognostic and predictive interpretation in TGCTs is strongest when biomarkers are interpreted across three levels: histogenesis and morphology, established clinicopathological risk systems, and emerging molecular/liquid-biopsy evidence. This synthesis shows that WHO 2022 morphology is still the diagnostic backbone, IGCCCG 2021 is still the clinical prognostic backbone, and miR-371a-3p is the most mature translational biomarker for non-teratomatous active disease. The evidence base reinforces this hierarchy but also shows that pre-analytical standardization, teratoma non-expression, and limited real-world sensitivity in some cohorts are decisive barriers to routine implementation and that no single layer is sufficient alone. The practical contribution of this review is therefore a graded, pathology-centered framework that separates established clinical markers from validated research markers and early exploratory candidates, helping avoid overstatement of single-marker evidence while identifying where prospective implementation studies would be most justified [12,13,22].

It is important to stress that the classical serum markers (AFP, β-hCG, and LDH) are central to staging and IGCCCG risk classification but have important limitations for relapse detection in CS1 disease and for accurate risk-group assignment if pre-orchidectomy (rather than pre-chemotherapy) levels are used [33,72]. In addition, the IGCCCG Update Consortium has substantially refined the survival estimates, with notable improvement in poor-prognosis NSGCT 5-year OS from 48% to 67% and identification of LDH ≥ 2.5 × ULN as an additional adverse factor in good-prognosis groups; these data should now replace the original 1997 figures in clinical and educational practice [12,13].

Among emerging biomarkers, miR-371a-3p has the strongest evidence base. In this regard, a combined analysis pooling several independent cohorts established that miR-371a-3p alone, rather than a full miR-302/367/371–373 panel, is sufficient for diagnostic use, with pooled sensitivity around 0.90 and specificity around 0.86 [26,32]. Moreover, the clinical utility of miR-371a-3p is consolidated by its rapid post-treatment normalization, its performance in residual-disease and RPLND pathology prediction [27,49,50], and the prospective CLIMATE data showing strong relapse-prediction performance in CS1 disease [85]. Follow-up data from the DRKS-00019223 study further support serum miR-371a-3p as a recurrence marker during stage I surveillance [133]. Equally important is the explicit recognition of two limitations: miR-371a-3p is typically negative in mature teratoma, and miR-371a-3p assay platforms, cut-offs, and normalization procedures must be standardized before integration into surveillance guidelines. In contrast, methylated RASSF1A cell-free DNA detection by droplet-digital PCR may partially address the teratoma blind spot when combined with miR-371a-3p [34,35,90]. In situ expression data also support the biological specificity of miR-371a-3p and miR-373-3p for malignant germ cell components, reinforcing why teratomatous disease continues to represent a liquid-biopsy limitation [134].

Currently, updated clinicopathological risk models are complementing the molecular markers. In this respect, the EAU 2023 three-tier model for CS1 seminoma [14,115] and the Danish stage I NSGCT analyses [118] provide externally validated frameworks for individualized surveillance versus adjuvant treatment and may, in the future, be augmented or partially replaced by post-orchidectomy miR-371a-3p. The interplay between IGCCCG-based metastatic risk and molecular biomarkers, for example very high LDH, teratoma in the primary tumor, immune microenvironmental features, and DNA-repair-rebalancing signatures, frames future precision-oncology research in TGCT [39,103,122].

### 4.3. Translational Challenges and Future Perspectives for Precision Oncology

Finally, several caveats deserve special emphasis. Although immune checkpoint molecules and systemic inflammatory indices have been intensively studied, they have not yet been translated into clinically actionable predictive biomarkers in TGCT, and immune-checkpoint inhibitors have shown only limited activity in heavily pre-treated refractory disease, while inflammatory indices are not independent of IGCCCG in multivariable models [112,113,114]. Additionally, despite the presence of activating KIT mutations in approximately 20–30% of seminomas, imatinib and related agents are ineffective because D816 activation-loop mutations determine primary resistance—a frequent misconception that should be corrected [67]. Moreover, the SOX2/SOX17 differential should be applied carefully in diagnostic practice, as follows: SOX17 (not SOX2) is the seminoma-associated pluripotency factor, whereas SOX2 marks embryonal carcinoma [31]. Finally, the central role of POU5F1/NANOG in driving cisplatin sensitivity—and the potential consequences of their loss during clonal evolution—is an active area of investigation with implications for resistance biomarker development [107].

The strengths of this review include systematic coverage of multiple marker classes (serum, immunohistochemical, transcription-factor, surface-receptor, microRNA, methylation, immune/microenvironmental, and clinicopathological), explicit evidence grading, integration of WHO 2022 nomenclature, the IGCCCG Update 2021, and contemporary EAU and SEOM-GG guidelines, comprising a clear distinction between established, validated, and emerging markers. Our study limitations include the heterogeneity of study design, marker platforms, cut-offs, and endpoints, which precluded a formal meta-analysis, and a degree of reliance on prior systematic reviews and meta-analyses for several quantitative estimates. As with any narrative synthesis, the selection of studies and the interpretation of conflicting findings introduce some degree of subjectivity, while rapid advances in liquid-biopsy assays and multi-omics may outpace the present synthesis.

## 5. Conclusions

Prognostic and predictive molecular markers in developmental and endocrine-sensitive testicular carcinogenesis are best understood as a continuum from developmental biology to clinical decision-making. Classical serum markers and the updated IGCCCG 2021 criteria are still the backbone of metastatic risk stratification, with LDH ≥ 2.5 × ULN added as an adverse factor and the explicit requirement to use pre-chemotherapy (not pre-orchidectomy) values.

Routine diagnostic IHC panels integrating OCT3/4, SALL4, D2-40, KIT, CD30, SOX17, SOX2, GPC3, and HNF1β, with PRAME as a useful complementary marker, provide solid subtype assignment under the WHO 2022 framework. miR-371a-3p is the biomarker closest to clinical implementation for non-teratomatous disease, with the caveat of teratoma negativity, which is partly addressed by combination with methylated RASSF1A cell-free DNA.

EAU 2023 risk models for CS1 seminoma and contemporary CS1 NSGCT analyses refine surveillance strategies, while prospective miR-371a-3p data may further support individualized management.

Future research should prioritize (i) harmonized PRISMA-based validation of biomarker panels across large international registries; (ii) integration of microRNA, methylated cfDNA, and multi-omics markers with established clinicopathological models; (iii) mechanistic studies linking specific molecular alterations to platinum resistance and late relapse; and (iv) careful assessment of how biomarker-driven strategies influence long-term survival and treatment-related toxicity in the predominantly young male TGCT population.

## Figures and Tables

**Table 1:** Evidence-graded summary of molecular markers in testicular germ cell tumors, distinguishing established clinical markers from validated research markers and emerging/unvalidated markers. Evidence levels are adapted from the Oxford Centre for Evidence-Based Medicine Levels of Evidence [28] (I = systematic review or RCT; II = large prospective cohort or pooled analysis; II-A = controlled study without randomization; II-B = quasi-experimental/cohort; III = retrospective cohort or translational data).

Marker/Panel	Category	Assay	Evidence Level	Status	Author/Years
AFP, β-hCG, LDH (serum)	classical serum	ELISA/ enzymatic	I	established clinical (IGCCCG, S-staging)	Beyer et al. 2021; Gillessen et al. 2021 [12,13]
OCT3/4, SALL4, SOX17, D2-40, CD117, CD30	IHC panel	IHC	II–A	established clinical (ISUP/WHO 2022)	Berney et al. 2022; Kao et al. 2020 [9,19]
PLAP, AP-2γ/TFAP2C, NANOG	IHC adjuncts	IHC	II–A/B	established (declining stand-alone use)	Chovanec et al. 2018; Siegmund et al. 2023 [1,15]
GPC3, HNF1β, FOXA2	yolk-sac IHC	IHC	II–A/B	established (GPC3); HNF1β/FOXA2 validated/emerging	Kao et al. 2020; Gallo et al. 2020 [19,29]
PRAME, SOX2	diagnostic IHC	IHC	II–B	validated research, approaching routine use	Cao et al. 2009; Jostes et al. 2019 [30,31]
miR-371a-3p (diagnosis)	circulating miRNA	RT-qPCR	II–A (pooled)	validated research; preclinical implementation	Dieckmann et al. 2019; Bagrodia et al. 2021 [18,32]
miR-371a-3p (CS1 MRD)	circulating miRNA	RT-qPCR	II–B (CLIMATE)	validated research; prospective validation ongoing	Heidenreich et al. 2023; Fankhauser et al. 2024 [33,34]
RASSF1A-methylated cfDNA	liquid biopsy	ddPCR	II–B	emerging (covers teratoma-only disease)	Lobo et al. 2021 [35]
ctDNA, cfDNA + N-glycan	liquid biopsy	NGS/ glycomics	III	emerging/ unvalidated	Ben-David et al. 2025; Krasić et al. 2025 [36,37]
i(12p), TCGA/100KGP signatures	genomic	karyotype/ WGS	II–A	established adjunct (GCNIS-derived tumors)	Litchfield et al. 2021; Cornish et al. 2024 [24,38]
NER deficiency, 53BP1, NHEJ markers	DNA- repair	IHC/molecular	III	emerging cisplatin- resistance biomarkers	Caggiano et al. 2021; Tamburello et al. 2026 [39,40]
RASSF1A/MGMT methylation (tissue)	epigenetic	bisulfite/MSP	II–B	emerging prognostic/ resistance	Esposito et al. 2025; Miranda-Gonçalves et al. 2021 [17,41]
m^6^A writers (VIRMA, METTL3)	epigenetic	RNA/IHC	III	emerging; preclinical	Esposito et al. 2025 [17]
PD-L1, VISTA, TIGIT	immune checkpoint	IHC	III	research; limited clinical utility to date	Cierna et al. 2016; Kunc et al. 2021 [42,43]
NLR, PLR, SII, HALP	systemic inflammation	CBC-derived	II–B/III	adjunct; not independent of IGCCCG	Kayar et al. 2025; Leško et al. 2025 [44,45]
miR-371a-3p (teratoma)	liquid biopsy	RT-qPCR	II	validated as a negative marker—teratoma not detected	Dieckmann et al. 2019; Lobo et al. 2021 [18,35]

IHC—immunohistochemistry.

**Table 2:** Mapping of evidence through July 2026 to study findings. The table separates biomarker findings suitable for narrative integration from data that are exploratory or insufficiently harmonized for de novo pooling.

Evidence Area	Key Extractable Finding	Study Findings	Author/Years
primary diagnosis/ small masses	miR-371a-3p is clinically useful but workflow- sensitive, with lower performance in unselected real-practice cohorts	miR-371a-3p is classified in the validated-research tier and is not yet used for routine universal screening	Chavarriaga et al. 2025; Matouskova et al. 2026; Seelemeyer et al. 2026 [46,47,48]
residual disease/RPLND	miR-371a-3p has high specificity for viable non-teratomatous tumor but weak teratoma detection; ctDNA also shows limited teratoma sensitivity	teratoma blind spot emphasized; cfDNA/ methylation approaches continue to be complementary and investigational	Kardoust Parizi et al. 2025; Dieckmann et al. 2026; Thakker et al. 2026 [27,49,50]
implementation	simplified plasma and serum M371 workflows show similar interim performance	pre-analytical standardization is a major barrier to clinical adoption	Heidenreich et al. 2026 [51]
cisplatin resistance and molecular evolution	EMT/SLUG, KDM6A/KDM6B, platinum-resistance alterations, p53/MYC/TFRC1 response, WEE1 inhibition, reciprocal loss of heterozygosity, TP53/MDM2 alterations, WNT activation, chromosome 3p25.3 gain, and clonal diversification are translational signals	resistance biomarkers are still emerging; integrated genomic studies support mechanistic risk stratification but no clinical meta-analysis or routine molecular-selection strategy is yet appropriate	Tamburello et al. 2026; Vieira Cardoso et al. 2024; Shokry et al. 2024; Cheng Sawa et al. 2024; Khan et al. 2026 [40,52,53,54,55]
immune/ transcriptomic markers	TIM-3/HAVCR2, LINC00152-linked T-cell exhaustion, TNFSF10, and immune-miRNA crosstalk expand the emerging immune-marker field	exploratory biomarkers; checkpoint and transcriptomic markers require prospective validation	Kunc et al. 2021; Zhang et al. 2023; Kim et al. 2026; Cao et al. 2026; Liu et al. 2026; Lopez-Saavedra et al. 2026 [43,56,57,58,59,60]
stage I risk models	contemporary risk models, and machine learning show moderate discrimination but require validation	EAU/DaTeCa/IGCCCG are still the baseline frameworks; molecular markers should augment rather than replace them	Strauch et al. 2026; Wagner et al. 2026 [61,62]

**Table 3:** Comparison of original IGCCCG 1997 and updated IGCCCG 2021 5-year progression-free survival (PFS) and overall survival (OS) estimates in metastatic NSGCTs and metastatic seminoma. The IGCCCG Update also identified LDH ≥ 2.5 × ULN, increasing age, and lung metastases as additional adverse factors in NSGCTs [12,13].

Prognostic Group	Histology	5 yr PFS 1997	5 yr PFS 2021	5 yr OS 1997	5 yr OS 2021
good	NSGCT	89%	89–90%	92%	96%
intermediate	NSGCT	75%	75–78%	80%	89%
poor	NSGCT	41%	54%	48%	67%
good	seminoma	82%	89%	86%	95%
intermediate	seminoma	67%	79%	72%	88%

**Table 4:** Classical serum tumor markers in TGCTs, showing tissue of origin, expected distribution by histological subtype, half-life, and prognostic role within S-staging and the updated IGCCCG (2021) classification.

Marker	Source/ Biology	Seminoma	Non-Seminomatous	Half-Life	Prognostic Role	Author/Years
AFP	fetal yolk-sac/liver glycoprotein	not produced by pure seminoma; elevation rules out pure seminoma	elevated in yolk-sac and embryonal carcinoma components	5–7 days	S-category and IGCCCG; rising or persistent values after orchidectomy signal occult disease	Gillessen et al. 2021; Dieckmann et al. 2019 [13,18]
β-hCG	syncytiotrophoblast-derived hormone	elevated in a minority of patients (hCG- producing seminoma with syncytiotrophoblast component)	high in choriocarcinoma and mixed non- seminomatous tumors	1–3 days	S-category and IGCCCG; very high pre-chemotherapy values associated with poor prognosis in NSGCTs	Beyer et al. 2021; Dieckmann et al. 2019 [12,18]
LDH	glycolytic enzyme; 12p-encoded isoforms	elevated in parallel with tumor burden	elevated in association with tumor burden	≈24 h	burden-reflective; LDH ≥ 2.5 × ULN identifies adverse subgroup within good-prognosis IGCCCG (2021)	Gillessen et al. 2021; Kao et al. 2020 [13,19]

**Table 5:** Updated immunohistochemical (IHC) panel for the diagnosis of testicular germ cell tumors by histological component. +, typically positive; −, typically negative; ±, variable/component-dependent. EC, embryonal carcinoma; YST, yolk-sac tumor; GCNIS, germ cell neoplasia in situ [9,19].

Marker	GCNIS	Seminoma	EC	YST	Choriocarcinoma	Teratoma
OCT3/4 (POU5F1)	+	+	+	−	−	−
NANOG	+	+	+	−	−	−
SALL4	+	+	+	+	+	±
SOX17	+	+	−	± (~73%)	−	−
SOX2	−	−	+	−	−	−
PLAP	+	+	±	−	−	−
D2-40 (podoplanin)	+	+	−	−	−	−
AP-2γ (TFAP2C)	+	+	+	−	−	−
CD117 (KIT)	+	+	−	± (~60%)	−	−
CD30	−	−	+	−	−	−
PRAME	−	+	−	±	−	−
GPC3	−	−	−	+	±	± (hepatoid subtype)
HNF1β	−	−	−	+	−	± (enteric subtype)
AFP (tissue)	−	−	−	± (patchy pattern)	−	−
β-hCG (tissue)	−	± (syncytiotrophoblast cells)	−	−	+	−
HPL	−	± (syncytiotrophoblast cells)	−	−	+	−
FOXA2 (emerging marker)	−	−	−	+	−	±

**Table 6:** EAU 2023 three-tier risk model for clinical stage I seminoma on active surveillance, derived from 1016 patients and externally validated in the TRISST randomized trial cohort. LVI, lymphovascular invasion; RTI, rete testis invasion [14,115].

Risk Group	Criteria	About 5-Year Relapse Rate
very low	tumor ≤ 2 cm, no RTI and no LVI	~8%
low/intermediate	tumor 2–5 cm, or RTI present, or single risk factor	~14–25%
high	tumor > 5 cm together with RTI and LVI	~44–45%

## Data Availability

No new data were created in this study. All data discussed are available in the publications and registries cited in the reference list.

## Supplementary Materials

The following supporting information can be downloaded at https://www.mdpi.com/article/10.3390/medsci14060633/s1, The completed PRISMA 2020 checklist [22] is available as Supplementary Materials.

## Abbreviations

AFP	alpha-fetoprotein
AJCC	American Joint Committee on Cancer
AP-2γ	activating enhancer-binding protein 2 gamma
AUC	area under the curve
BEP	bleomycin, etoposide, cisplatin
β-hCG	beta human chorionic gonadotropin
Ca	carcinoma
CAR-T	chimeric antigen receptor T-cell therapy
CD30	cluster of differentiation 30
CD117	cluster of differentiation 117/KIT receptor
CEBM	Centre for Evidence-Based Medicine
cfDNA	cell-free DNA
chorio	choriocarcinoma
CLDN6	claudin-6
Cq	quantification cycle
CS1	clinical stage I
ctDNA	circulating tumor DNA
D2-40	podoplanin immunohistochemical marker
ddPCR	droplet digital polymerase chain reaction
EAU	European Association of Urology
EC	embryonal carcinoma
ELISA	enzyme-linked immunosorbent assay
FOXA2	forkhead box A2
GCNIS	germ cell neoplasia in situ
GCT	germ cell tumor
GPC3	glypican-3
HALP	haemoglobin–albumin–lymphocyte–platelet score
HERV	human endogenous retrovirus
HNF1β	hepatocyte nuclear factor 1 beta
HPL	human placental lactogen
HR	hazard ratio
IGCCCG	International Germ Cell Cancer Collaborative Group
IHC	immunohistochemistry
ISUP	International Society of Urological Pathology
KIT	KIT proto-oncogene receptor tyrosine kinase
LDH	lactate dehydrogenase
LIN28	lin-28 homolog (pluripotency-associated RNA-binding protein)
LVI	lymphovascular invasion
miR	microRNA
miR-302/367	microRNA-302/367 cluster
miR-371a-3p	microRNA-371a-3p
miR-373-3p	microRNA-373-3p
MRD	minimal residual disease
NANOG	Nanog homeobox
NGS	next-generation sequencing
NLR	neutrophil-to-lymphocyte ratio
NPV/PPV	negative/positive predictive value
NSGCT	non-seminomatous germ cell tumor
OCT3/4	octamer-binding transcription factor 3/4
OGCT	ovarian germ cell tumor
OS	overall survival
PD-L1	programmed death-ligand 1
PFS	progression-free survival
PGC	primordial germ cell
PLAP	placental alkaline phosphatase
PLR	platelet-to-lymphocyte ratio
POU5F1	POU class 5 homeobox 1
PRAME	preferentially expressed antigen in melanoma
RASSF1A	Ras association domain family member 1 isoform A
RCT	randomized controlled trial
RPLND	retroperitoneal lymph node dissection
RTI	rete testis invasion
SEER	Surveillance, Epidemiology, and End Results
SII	systemic immune–inflammation index
SOX2	SRY-box transcription factor 2
SOX17	SRY-box transcription factor 17
TCGA	The Cancer Genome Atlas
TDS	testicular dysgenesis syndrome
TF	transcription factor
TFAP2C	transcription factor AP-2 gamma
TGCT	testicular germ cell tumor
TME	tumor microenvironment
ULN	upper limit of normal
VISTA	V-domain Ig suppressor of T-cell activation
WHO	World Health Organization
YST	yolk sac tumor
