# Supplementary material for: From Germ Cell Histogenesis to Precision Risk Stratification: Prognostic and Predictive Molecular Markers in Testicular Germ Cell Tumors—A Systematic Review
Source: Med Sci (Basel). 2026 Oct 2;14(6):633. doi: 10.3390/medsci14060633 (PMC13707152; doi:10.3390/medsci14060633)
Supplement: Supplementary file 1 [file medsci-14-00633-s001.zip › medsci-4528464-supplementary.pdf]

PRISMA Documentation

From Germ-Cell Histogenesis to Precision Risk Stratification: Prognostic and Predictive Molecular Markers in Testicular Germ Cell Tumors — A Systematic Review

Andrei Bogdan Cutitaru [1,], Irina Draga Caruntu [1], Raluca Anca Balan [1], Carmen Solcan [2] and Cornelia Amalinei [1]*

Department of Morphofunctional Sciences I, Grigore T. Popa University of Medicine and Pharmacy Iasi, 700115 Iasi, Romania; Faculty of Veterinary Medicine, Ion Ionescu de la Brad University of Life Sciences Iasi, Aleea Mihail Sadoveanu No. 3, 700490 Iasi, Romania.

Search coverage: database inception to 6 July 2026.

This document consolidates the literature-search, de-duplication, screening, eligibility, inclusion, and citation-mapping audit trail for the systematic review.

1. Review question and PICO framework

Review question: Which molecular, immunohistochemical, serum, and liquid-biopsy markers provide prognostic or predictive information in TGCTs beyond conventional clinicopathological assessment?

PICO element	Definition applied
Population (P)	Patients with seminomatous or non-seminomatous TGCT or germ cell neoplasia in situ (GCNIS), together with experimental models of developmental and endocrine-sensitive testicular carcinogenesis.
Intervention / exposure (I)	Assessment of molecular, immunohistochemical, serum, or liquid-biopsy biomarkers, including transcription factors, surface markers, genomic signatures, epigenetic signatures, and microRNA signatures.
Comparator (C)	Conventional clinicopathological factors, standard serum markers (AFP, β-hCG, LDH), or absent/low expression of the candidate biomarker; healthy or benign testicular tissue in translational studies.
Outcomes (O)	Diagnostic classification, stage at presentation, progression-free survival, relapse risk, overall survival, therapy response, and IGCCCG risk-group assignment.

2. Eligibility criteria

Domain	Eligible	Not eligible
Population	TGCT, including seminoma, non-seminoma, GCNIS, and models of testicular germ-cell carcinogenesis.	Non-testicular or non-germ-cell tumors; testicular tumors of non-germ-cell origin when reported in isolation.
Marker/exposure	Molecular, IHC, serum, genomic, epigenetic, microRNA, and liquid-biopsy markers.	Reports without any marker measurement.
Outcome	Diagnostic, prognostic, predictive, monitoring, or risk-model outcomes.	Therapy-efficacy outcomes without a marker-outcome relationship.
Source type	Primary clinical and translational studies, systematic reviews and meta-analyses, guidelines, classification sources, and registry or modelling sources.	Records with no extractable marker or outcome data.
Language / availability	Records readable in the original language or in translation.	Records without accessible full text or usable translation.

3. Information sources

The search was conducted as one continuous process covering database inception to 6 July 2026. MEDLINE/PubMed, Embase, and Web of Science Core Collection were the primary bibliographic sources. The Cochrane Library was used as a cross-check for Cochrane Reviews, Cochrane Protocols, and CENTRAL/Trials records. Reference lists of eligible articles, key

reviews, guidelines, classification sources, registry-based studies, and IGCCCG Consortium publications were also reviewed for supplementary source checking.

4. Search strategies as executed

4.1 Core concept sets

- Disease terms: testicular germ cell tumor / tumour; testicular neoplasms; seminoma; nonseminoma; non-seminoma; germ cell neoplasia in situ; GCNIS; TGCT.
- Marker and mechanism terms: biomarker; marker; prognostic; predictive; molecular; serum marker; microRNA; miR-371a-3p; M371; OCT4; POU5F1; NANOG; SALL4; SOX17; KIT; RASSF1A; liquid biopsy; cell-free DNA; cfDNA; methylation; immune microenvironment; cisplatin resistance; risk model; nomogram.

4.2 MEDLINE/PubMed

((testicular germ cell tumor*[tiab] OR testicular germ cell tumour*[tiab] OR testicular neoplasms[mh] OR seminoma[tiab] OR nonseminoma[tiab] OR non-seminoma[tiab]) AND (prognos*[tiab] OR predict*[tiab] OR biomarker*[tiab] OR marker*[tiab] OR molecular[tiab]))

Coverage applied: database inception to 6 July 2026. The displayed count used for PRISMA identification was 4,921 records. All results were exported in a single PubMed Citation Manager file and verified by PMID count.

4.3 Embase

('testicular germ cell tumor'/exp OR 'testicular germ cell tumor' OR 'seminoma'/exp OR 'seminoma' OR 'nonseminoma'/exp OR 'nonseminoma' OR 'germ cell neoplasm'/exp OR 'testicular cancer'/exp OR 'testicular cancer' OR tgct OR 'gcnis'/exp OR gcnis OR 'germ cell neoplasia in situ'/exp OR 'germ cell neoplasia in situ') AND ('biomarker'/exp OR 'biomarker' OR 'prognosis'/exp OR 'prognosis' OR 'predictive marker'/exp OR 'predictive marker' OR 'mir-371a-3p' OR 'microrna'/exp OR 'microrna' OR 'oct4' OR 'pou5f1' OR 'nanog' OR 'sall4'/exp OR 'sall4' OR 'sox17' OR 'kit'/exp OR 'kit' OR 'rassf1a' OR 'liquid biopsy'/exp OR 'liquid biopsy' OR 'cell free dna'/exp OR 'cell free dna' OR 'cfdna' OR 'dna methylation'/exp OR 'dna methylation' OR 'tumor microenvironment'/exp OR 'tumor microenvironment' OR 'cisplatin resistance'/exp OR 'cisplatin resistance' OR 'risk model'/exp OR 'risk model' OR 'nomogram'/exp OR 'nomogram')

Search settings: map to preferred Emtree term; search also as free text in all fields; explode using narrower Emtree terms; broad search setting. The major-focus option was not selected. No language, publication-type, or other limits were applied.

4.4 Web of Science Core Collection

TS=(("testicular germ cell" OR seminoma OR nonseminoma OR TGCT OR GCNIS OR "germ cell neoplasia in situ") AND (biomarker OR prognostic OR predictive OR "miR-371a-3p" OR microRNA OR OCT4 OR POU5F1 OR NANOG OR SALL4 OR SOX17 OR KIT OR RASSF1A OR "liquid biopsy" OR cfDNA OR methylation OR "immune microenvironment" OR "cisplatin resistance" OR nomogram))

The query was run in the Topic field with all Web of Science Core Collection editions selected across all years and no filters or limits applied. The displayed count was 2,276 records.

4.5 Cochrane Library

(testicular germ cell tumor OR seminoma OR nonseminoma) AND (biomarker OR molecular marker OR miR-371a-3p OR microRNA OR cisplatin resistance)

The cross-check identified 0 Cochrane Reviews, 0 Cochrane Protocols, and 36 CENTRAL/Trials records.

5. Identification counts

Source	Records identified	Evidence basis
MEDLINE/PubMed	4,921	Single PubMed Citation Manager file; 4,921 records by PMID count.
Embase	12,739	Two contiguous RIS batches; 12,739 parsed records, 12,739 TY markers, 12,739 ER markers, and 12,739 unique U2 identifiers.

Web of Science Core Collection	2,276	Three RIS batches totaling 2,276 records by ER markers.
Database total before cross-database de-duplication	19,936	Arithmetic sum of database-specific records before duplicate removal.

6. Cochrane cross-check

Cochrane category	Records
Cochrane Reviews	0
Cochrane Protocols	0
CENTRAL/Trials	36

The 36 CENTRAL/Trials records were retained as a count-only cross-check and were not imported into the de-duplicated bibliographic pool because record-level export was not available for this set.

7. De-duplication method and counts

De-duplication was applied to MEDLINE/PubMed, Embase, and Web of Science Core Collection records. Cochrane records were excluded from the de-duplicated pool because the cross-check was count-only.

Deduplication criterion
normalized DOI exact match with matching normalized title
PMID exact match
Embase PUI exact match
Web of Science accession exact match
normalized title + year + first author exact match

Measure	Count
Records imported for de-duplication	19,936
Duplicate groups	2,656
Duplicates removed	3,240
Unique records for title/abstract screening	16,696

Primary duplicate reason	Duplicates removed
normalized DOI exact match with matching normalized title	2,854
PMID exact match	196
normalized title + year + first author exact match	190

Duplicate group size	Groups
2	2,078
3	572
4	6

Source combination	Duplicate groups
PubMed/MEDLINE; Embase; Web of Science Core Collection	558
PubMed/MEDLINE; Web of Science Core Collection	647
PubMed/MEDLINE; Embase	1,142
Embase; Web of Science Core Collection	181
Embase	112
Web of Science Core Collection	16

8. Title/abstract screening

Screening was conservative: ambiguous records plausibly involving TGCT, GCNIS, testicular germ-cell carcinogenesis, and a biomarker, molecular, IHC, serum, liquid-biopsy, prognostic, predictive, monitoring, resistance, or risk-model concept were advanced to full-text assessment. Full-text eligibility was not determined at this stage.

Measure	Count
Unique records screened	16,696
Records excluded at title/abstract screening	12,851
Records advanced to full-text assessment	3,845
Records with abstract available	9,569
Records screened with title and bibliographic metadata only	7,127

Primary title/abstract exclusion reason	Records excluded
Outside TGCT/testicular germ-cell population	8,810
No biomarker/molecular/risk-marker concept	1,691
General therapy/management without biomarker outcome	776
Non-human/non-TGCT-only without translational bridge	50
Publication type with insufficient extractable marker/outcome content	1,300
Duplicate remnant after de-duplication	220
Insufficient bibliographic information	4

9. Full-text eligibility assessment

Measure	Count
Reports sought for retrieval	3,845
Reports not retrieved	958
Reports assessed for eligibility	2,887
Full-text reports excluded with primary reasons	2,807
Database-screened reports included	80

Primary reason	Count type	Count
inaccessible full text/unusable language	reports not retrieved	958
expression-only/no clinical or diagnostic outcome	full-text reports excluded	1,118
no extractable marker-outcome data	full-text reports excluded	891
wrong tumor type	full-text reports excluded	251
publication type insufficient	full-text reports excluded	146
therapy-only without marker endpoint	full-text reports excluded	136
already represented by retained systematic review/meta-analysis where appropriate	full-text reports excluded	124
wrong population	full-text reports excluded	94
non-human/lab-only without TGCT translational bridge	full-text reports excluded	47

10. Final inclusion and supplementary source checking

Measure	Count
Database-screened reports included	80
Supplementary source reports included	46
Final reports included in review	126
Reports included in biomarker evidence synthesis	124
Methodological reporting sources	2

The final inclusion count equals the database-screened included reports plus the supplementary source reports. The final evidence synthesis count excludes the two methodological reporting sources from biomarker evidence synthesis.

Source route	Reports
database screened candidate	80
supplementary source checking	46

11. Citation-to-evidence map summary

The citation-to-evidence map contains 126 numbered entries aligned with the final manuscript reference list. Entries provide brief descriptive labels for the evidence supporting each thematic section rather than quotations.

Source type	Reports
primary clinical study	51
narrative review	30
translational/laboratory study	13
classification/reference text	9
systematic review or meta-analysis	8
guideline	7
registry/epidemiology	6
methodological standard	2

Evidence-map section	Reports
Introduction	25
microRNA/liquid biopsy	21
Cisplatin resistance	15
Risk models and clinicopathology	13
Guidelines/registries	11
Immune microenvironment	9
Discussion	8
Pluripotency/IHC markers	9
Genomics/transcriptomics	5
Yolk-sac and trophoblastic markers	3
cfDNA/methylation	3
Methods	2
Serum markers	2

11.1 Citation-to-evidence map

The table below provides the complete citation-to-evidence map for the 126 numbered references aligned with the final manuscript reference list.

Ref.	Source	Data point / concept used in the manuscript	Section of use	Source type
**1**	Chovanec et al., 2018	Emerging prognostic biomarkers beyond established practice	Introduction	narrative review
**2**	Albany et al., 2018	High-volume/SEER survival outcomes in metastatic GCT	Introduction	registry/epidemiolog y
**3**	Hanna et al., 2022	TGCT epidemiology and incidence overview	Introduction	classification/refere nce text
**4**	Lobo et al., 2023	Molecular biomarkers with clinical application in testicular cancer	Discussion	narrative review
**5**	Oliveira-Lopes et al., 2025	Testicular germ cell tumours and molecular biomarkers	Discussion	narrative review
**6**	Mitchell et al., 2014	GCNIS heterogeneous protein expression and invasion	Introduction	primary clinical study
**7**	Oosterhuis and Looijenga, 2010	GCNIS precursor biology and associated cancers	Introduction	narrative review
**8**	Shen et al., 2018	TGCT developmental carcinogenesis and GCNIS precursor	Introduction	classification/refere nce text
**9**	Berney et al., 2022	WHO 5th edition TGCT classification	Introduction	classification/refere nce text
**10**	Moch et al., 2022	WHO 2022 TGCT classification and GCNIS	Introduction	classification/refere

Ref.	Source	Data point / concept used in the manuscript	Section of use	Source type
		terminology		nce text
**11**	Bremmer et al., 2023	WHO 2022 TGCT classification revisions	Introduction	classification/reference text
**12**	Beyer et al., 2021	IGCCCG 2021 guidance	Guidelines/registries	guideline
**13**	Gillessen et al., 2021	IGCCCG 2021 evidence for metastatic NSGCT outcomes	Guidelines/registries	primary clinical study
**14**	Boormans et al., 2023	EAU germ cell tumor guideline risk groups	Guidelines/registries	guideline
**15**	Siegmund et al., 2023	Diagnostic tissue-based biomarkers in testicular tumors	Pluripotency/IHC markers	narrative review
**16**	Arranz Arija et al., 2024	SEOM-GG TGCT management guideline	Guidelines/registries	guideline
**17**	Esposito et al., 2025	TGCT biomarkers and therapeutic targets overview	Introduction	narrative review
**18**	Dieckmann et al., 2019	Serological and IHC TGCT markers	Introduction	narrative review
**19**	Kao et al., 2020	ISUP molecular pathology consensus	Guidelines/registries	classification/reference text
**20**	National Cancer Institute (SEER), 2025	TGCT incidence and mortality statistics	Introduction	registry/epidemiology
**21**	Patrikidou et al., 2026	EAU 2026 testicular cancer guideline	Guidelines/registries	guideline
**22**	Page et al., 2021	PRISMA 2020 reporting guideline	Methods	methodological standard
**23**	Leão et al., 2021	Circulating microRNAs as serum biomarkers	microRNA/liquid biopsy	systematic review or meta-analysis
**24**	Litchfield et al., 2021	TGCT biomarker risk stratification and treatment prediction	Discussion	systematic review or meta-analysis
**25**	Zhao et al., 2021	MicroRNA diagnostic performance in TGCT	microRNA/liquid biopsy	systematic review or meta-analysis
**26**	Seales et al., 2024	MiR-371a-3p as promising TGCT serum marker	microRNA/liquid biopsy	narrative review
**27**	Kardoust Parizi et al., 2025	MiR-371a-3p predicts viable residual tumor at RPLND	microRNA/liquid biopsy	systematic review or meta-analysis
**28**	OCEBM Levels of Evidence Working Group, 2011	Oxford CEBM levels of evidence	Methods	methodological standard
**29**	Gallo et al., 2020	HNF1β as sensitive YST marker	Yolk-sac and trophoblastic markers	primary clinical study
**30**	Cao et al., 2009	SALL4 diagnostic marker in testicular GCT	Pluripotency/IHC markers	primary clinical study
**31**	Jostes et al., 2019	SOX17 replaces SOX2 in seminoma/GCNIS	Pluripotency/IHC markers	primary clinical study
**32**	Bagrodia et al., 2021	MiR-371a-3p sufficient for TGCT diagnosis	microRNA/liquid biopsy	primary clinical study
**33**	Heidenreich et al., 2023	Pre-chemotherapy markers for IGCCCG assignment	Guidelines/registries	primary clinical study
**34**	Fankhauser et al., 2024	MiR-371a-3p relapse surveillance cut-off	microRNA/liquid biopsy	primary clinical study
**35**	Lobo et al., 2021	Serum marker sensitivity for active TGCT	Serum markers	primary clinical study
**36**	Ben-David et al., 2025	Tumour-informed ctDNA in TGCT	cfDNA/methylation	primary clinical study
**37**	Krasić et al., 2025	Multi-analyte liquid biopsy for NSGCT	microRNA/liquid biopsy	primary clinical study
**38**	Cornish et al., 2024	Genomic landscape of adult TGCT	Genomics/transcriptomics	primary clinical study
**39**	Caggiano et al., 2021	DNA repair pathway rebalancing in cisplatin resistance	Cisplatin resistance	translational/laboratory study
**40**	Tamburello et al., 2026	WEE1 inhibition overcomes cisplatin resistance	Cisplatin resistance	translational/laboratory study

Ref.	Source	Data point / concept used in the manuscript	Section of use	Source type
**41**	Miranda-Gonçalves et al., 2021	VIRMA-mediated cisplatin resistance	Cisplatin resistance	translational/laboratory study
**42**	Cierna et al., 2016	PD-L1 prognostic value in TGCT	Immune microenvironment	primary clinical study
**43**	Kunc et al., 2021	VISTA plus PLR predicts worse advanced TGCT outcome	Immune microenvironment	primary clinical study
**44**	Kayar et al., 2025	HALP score for TGCT risk stratification	Risk models and clinicopathology	primary clinical study
**45**	Leško and Mego, 2025	Post-orchiectomy SII associated with tumor characteristics	Risk models and clinicopathology	primary clinical study
**46**	Chavarriaga et al., 2025	MiR-371a-3p liquid biomarker for small masses	microRNA/liquid biopsy	primary clinical study
**47**	Matouskova et al., 2026	Parallel miR-371a-3p and cfDNA liquid biopsy	microRNA/liquid biopsy	primary clinical study
**48**	Seelemeyer et al., 2026	MiR-371a assay in small testicular masses	microRNA/liquid biopsy	primary clinical study
**49**	Dieckmann et al., 2026	MiR-371a-3p predicts RPLND histology	microRNA/liquid biopsy	primary clinical study
**50**	Thakker et al., 2026	CtDNA predicts retroperitoneal histology	microRNA/liquid biopsy	primary clinical study
**51**	Heidenreich et al., 2026	MiR-371a-3p clinical routine validation	microRNA/liquid biopsy	narrative review
**52**	Vieira Cardoso et al., 2024	SLUG/EMT in cisplatin resistance	Cisplatin resistance	translational/laboratory study
**53**	Shokry et al., 2024	KDM6A/KDM6B inhibition sensitizes TGCT to cisplatin	Cisplatin resistance	translational/laboratory study
**54**	Cheng Sawa et al., 2024	Driver mutations linked to platinum sensitivity	Cisplatin resistance	primary clinical study
**55**	Khan et al., 2026	Cisplatin transcriptomic response in TGCT resistance	Cisplatin resistance	translational/laboratory study
**56**	Zhang et al., 2023	Single-cell TGCT microenvironment and immune subtypes	Discussion	primary clinical study
**57**	Kim et al., 2026	HAVCR2/TIM-3 favorable prognostic marker in seminoma	Immune microenvironment	translational/laboratory study
**58**	Cao et al., 2026	LINC00152-high TGCT cells linked to poor prognosis	Genomics/transcriptomics	translational/laboratory study
**59**	Liu et al., 2026	TNFSF10 prognostic and immunotherapy marker	Immune microenvironment	translational/laboratory study
**60**	Lopez-Saavedra et al., 2026	MicroRNAs and immunotherapy in TGCT	Immune microenvironment	narrative review
**61**	Strauch et al., 2026	Stage I seminoma risk models	Risk models and clinicopathology	primary clinical study
**62**	Wagner et al., 2026	Machine-learning relapse prediction in stage I TGCT	Risk models and clinicopathology	primary clinical study
**63**	De Jong, 2007	OCT4 biology and diagnostic utility	Pluripotency/IHC markers	classification/reference text
**64**	Skakkebæk et al., 2024	Cryptorchidism and endocrine-disrupting chemicals as TGCT risk factors	Introduction	narrative review
**65**	Ciarloni et al., 2025	TDS and endocrine disruptor risk factors	Introduction	narrative review
**66**	Kristensen et al., 2008	Microenvironmental origins of pluripotent TGCT	Introduction	narrative review
**67**	Colopi et al., 2024	RAS/MAPK signaling in TGCT	Introduction	narrative review
**68**	Steliarova-Foucher et al., 2019	International childhood/adolescent TGCT incidence rates	Guidelines/registries	registry/epidemiology
**69**	Cancer Research UK, 2023	TGCT incidence and survival statistics	Introduction	registry/epidemiology
**70**	Patrikidou et al., 2023	EAU 2023 TGCT guideline update	Guidelines/registries	guideline
**71**	Ghazarian et al., 2024	Regional SEER variation in stage III nonseminoma	Guidelines/registries	registry/epidemiology
**72**	Hamilton et al., 2023	Pre-chemotherapy markers for IGCCCG classification	Risk models and clinicopathology	primary clinical study

Ref.	Source	Data point / concept used in the manuscript	Section of use	Source type
**73**	Gilligan et al., 2010	Serum tumor marker uses in adult male GCT	Serum markers	guideline
**74**	Kaldany et al., 2024	Current evolving TGCT biomarkers overview	Introduction	narrative review
**75**	Rajpert-De Meyts et al., 2015	Diagnostic IHC markers for GCNIS and TGCT	Pluripotency/IHC markers	narrative review
**76**	de Jong et al., 2008	SOX2 negative in seminoma; embryonal carcinoma marker	Pluripotency/IHC markers	primary clinical study
**77**	Kotthoff et al., 2025	SOX17-driven yolk-sac differentiation and cisplatin resistance	Cisplatin resistance	translational/laboratory study
**78**	Zhou et al., 2022	PRAME seminoma marker sensitivity/specificity	Pluripotency/IHC markers	primary clinical study
**79**	Nathanson et al., 2009	KITLG 5q31.3 TGCT susceptibility locus	Genomics/transcriptomics	primary clinical study
**80**	Yao et al., 2023	Seminoma KRAS/NRAS mutations	Genomics/transcriptomics	translational/laboratory study
**81**	Kemmer et al., 2004	KIT mutations common in seminoma	Genomics/transcriptomics	primary clinical study
**82**	Zynger et al., 2008	GPC3 expression in YST	Yolk-sac and trophoblastic markers	primary clinical study
**83**	Ulbright et al., 1997	Trophoblastic tumors and hPL/β-hCG positivity	Yolk-sac and trophoblastic markers	primary clinical study
**84**	Belge et al., 2023	MiR-371a-3p serum biomarker in TGCT	microRNA/liquid biopsy	narrative review
**85**	Tran et al., 2026	Post-orchidectomy miR-371a-3p predicts relapse	microRNA/liquid biopsy	primary clinical study
**86**	Janssen et al., 2026	Liquid biopsy methylation biomarkers for GCT subtypes	cfDNA/methylation	primary clinical study
**87**	Steenbock et al., 2026	MiR-371a-3p monitoring during BEP therapy	microRNA/liquid biopsy	primary clinical study
**88**	Myklebust et al., 2022	MicroRNAs in teratoma differentiation	microRNA/liquid biopsy	translational/laboratory study
**89**	Thor et al., 2024	MiR-371a-3p predicts viable tumor at RPLND	microRNA/liquid biopsy	primary clinical study
**90**	Garrett et al., 2025	Polygenic TGCT susceptibility model	Discussion	systematic review or meta-analysis
**91**	Spinella et al., 2021	Genome-wide hypomethylation and cisplatin hypersensitivity in seminoma	Discussion	narrative review
**92**	Nicu et al., 2022	TGCT epigenetics and methylation	Discussion	narrative review
**93**	Nicu et al., 2024	TGCT DNA methylation and immune relationship	Discussion	narrative review
**94**	Acquaviva et al., 2023	Salvage therapy biomarkers in TGCT	Cisplatin resistance	narrative review
**95**	Hansen et al., 2023	Neddylation contributes to cisplatin resistance	Cisplatin resistance	primary clinical study
**96**	Zhan et al., 2025	Cisplatin resistance mechanisms and therapeutic targets	Cisplatin resistance	narrative review
**97**	Gutekunst et al., 2011	P53 hypersensitivity mediates cisplatin responsiveness	Cisplatin resistance	primary clinical study
**98**	Gutekunst et al., 2013	OCT4-mediated NOXA and cisplatin hypersensitivity	Cisplatin resistance	primary clinical study
**99**	Parola et al., 2024	PARP inhibitors and cisplatin-resistance context in TGCT	Cisplatin resistance	narrative review
**100**	Wheeler et al., 2025	Epigenomic drivers of histology-specific TGCT	Cisplatin resistance	translational/laboratory study
**101**	Fankhauser et al., 2015	Frequent PD-L1 expression in TGCT	Immune microenvironment	primary clinical study
**102**	Fankhauser et al., 2018	Systemic inflammatory markers prognostic in metastatic TGCT	Risk models and clinicopathology	primary clinical study
**103**	Ozgur et al., 2024	Limited activity of immune checkpoint inhibitors	Immune microenvironment	systematic review or meta-analysis
**104**	Bleve et al., 2022	Inflammatory biomarkers for outcome prediction	Immune microenvironment	systematic review or meta-analysis
**105**	Clark et al., 2024	NLR prognostic value in TGCT	Immune	systematic review

Ref.	Source	Data point / concept used in the manuscript	Section of use	Source type
			microenvironment	or meta-analysis
**106**	Huddart et al., 2024	TRISST external validation of EAU seminoma risk model	Risk models and clinicopathology	primary clinical study
**107**	Aparicio et al., 2014	Stage I seminoma relapse nomogram based on tumor size and RTI	Risk models and clinicopathology	primary clinical study
**108**	Albers et al., 2003	LVI predicts relapse in CS1 NSGCT	Risk models and clinicopathology	primary clinical study
**109**	Wagner et al., 2024	Danish nationwide CS1 non-seminoma relapse-risk model	Risk models and clinicopathology	primary clinical study
**110**	European Association of Urology, 2026	EAU staging and classification guidance	Guidelines/registries	guideline
**111**	Smith et al., 2025	Relapse-detection methods in stage I active surveillance	Risk models and clinicopathology	narrative review
**112**	Gillessen et al., 2019	Tumour-marker gradients in intermediate-prognosis NSGCT	Risk models and clinicopathology	registry/epidemiology
**113**	Necchi et al., 2024	Teratoma in primary tumor affects survival	Risk models and clinicopathology	primary clinical study
**114**	Maharjan et al., 2026	GCNIS reprogramming outside spermatogonial niche	Pluripotency/IHC markers	primary clinical study
**115**	Conduit et al., 2026	CLDN6 in GCNIS and non-teratomatous TGCT	Pluripotency/IHC markers	primary clinical study
**116**	Sköld et al., 2024	Testicular and ovarian GCT common features, different prognosis	Introduction	narrative review
**117**	Fichtner et al., 2024	TGCT pathogenesis and pathological diagnostics	Introduction	classification/reference text
**118**	Liliac et al., 2012	Ovarian tumor histogenesis contrast	Introduction	narrative review
**119**	Oosterhuis et al., 2019	Developmental perspective on TGCT carcinogenesis	Introduction	narrative review
**120**	Reuter, 2005	I(12p) genomic hallmark of post-pubertal TGCT	Introduction	classification/reference text
**121**	Dieckmann and Belge, 2024	MiR-371a-3p diagnostic and monitoring value	microRNA/liquid biopsy	narrative review
**122**	Dantkale et al., 2024	Ovarian germ cell tumor diagnosis and treatment review	Introduction	narrative review
**123**	Travis et al., 2024	AYA germ cell tumor epidemiology and genomics	Introduction	narrative review
**124**	Dieckmann et al., 2024	MiR-371a-3p recurrence marker in stage I surveillance	microRNA/liquid biopsy	primary clinical study
**125**	Lobo et al., 2021	Hypermethylated RASSF1A ddPCR plus miR-371a-3p panel	cfDNA/methylation	primary clinical study
**126**	Mørup et al., 2026	MiR-371a-3p/miR-373-3p in situ expression	microRNA/liquid biopsy	primary clinical study

12. PRISMA flow data table

Flow field	Count
Records identified from MEDLINE/PubMed	4,921
Records identified from Embase	12,739
Records identified from Web of Science Core Collection	2,276
Database records imported for de-duplication	19,936
Duplicates removed	3,240
Records screened	16,696
Records excluded at title/abstract screening	12,851
Reports sought for retrieval	3,845
Reports not retrieved	958
Reports assessed for eligibility	2,887
Full-text reports excluded	2,807
Database-screened reports included	80

Supplementary source reports included	46
Final reports included	126
Reports included in biomarker evidence synthesis	124
Methodological reporting sources	2

13. PRISMA-style flow figure

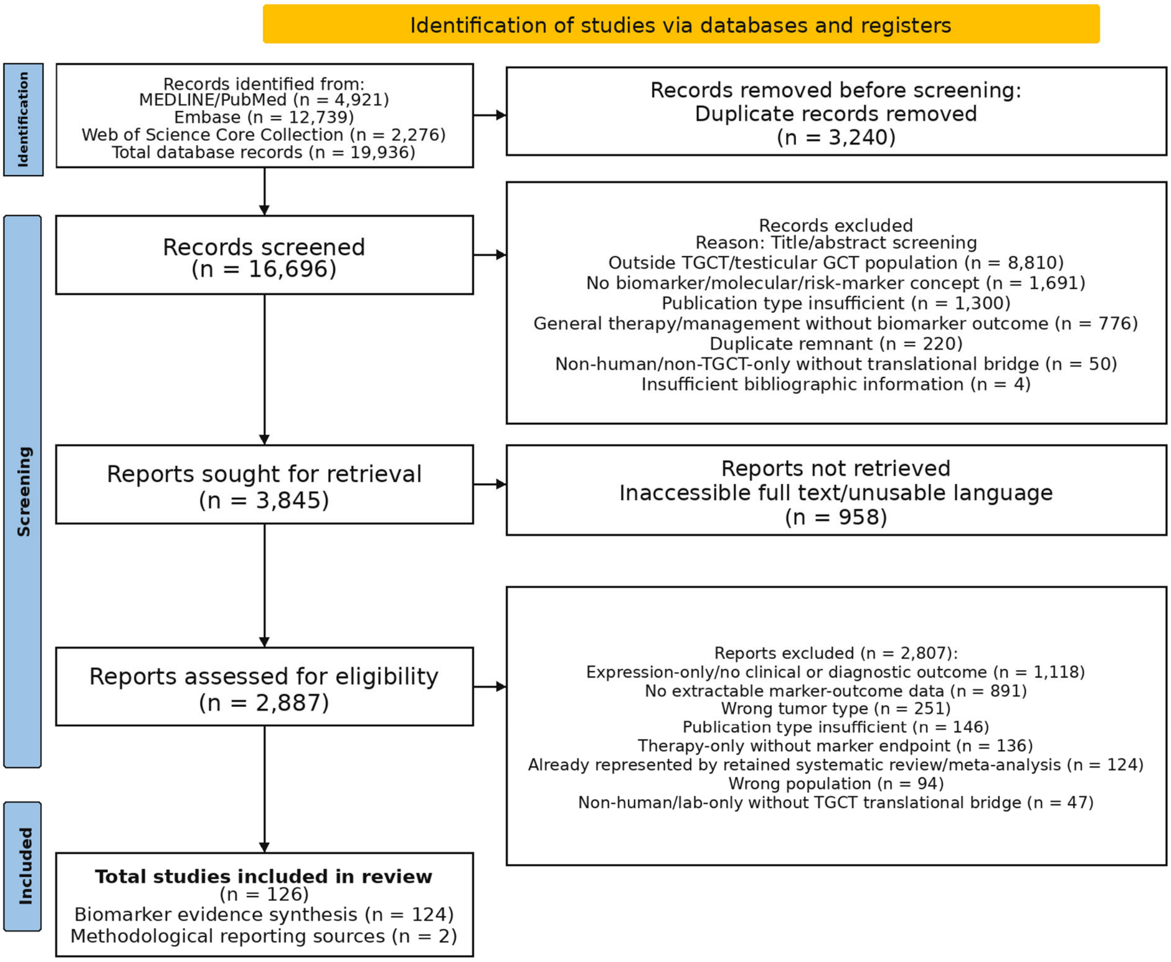

Figure S1. PRISMA 2020 flow diagram of the study selection process.

Appendix A. Cochrane Library count capture

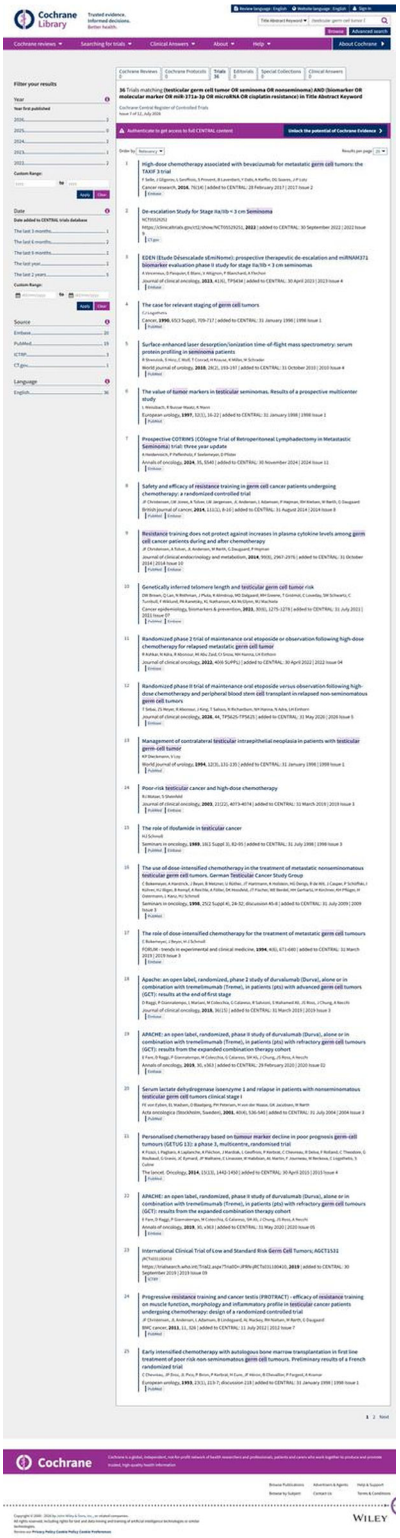

Figure A. Cochrane Library count capture.
